# MXene-Derived Potassium-Preintercalated Bilayered Vanadium Oxide Nanostructures for Cathodes in Nonaqueous K-Ion Batteries

**DOI:** 10.1021/acsanm.5c00175

**Published:** 2025-04-07

**Authors:** Timofey Averianov, Xinle Zhang, Ryan Andris, Daniel Olds, Michael J. Zachman, Ekaterina Pomerantseva

**Affiliations:** † Materials Electrochemistry Group, Department of Materials Science and Engineering, Drexel University, Philadelphia, Pennsylvania 19104, United States; ‡ National Synchrotron Light Source II, Brookhaven National Laboratory, Upton, New York 11973, United States; § Center for Nanophase Materials Sciences, Oak Ridge National Laboratory, Oak Ridge, Tennessee 37831, United States

**Keywords:** chemically preintercalated bilayered vanadium oxides, MXene-derived oxides, MAX phase etchant composition, K-ion batteries, morphological stabilization, charge storage mechanism

## Abstract

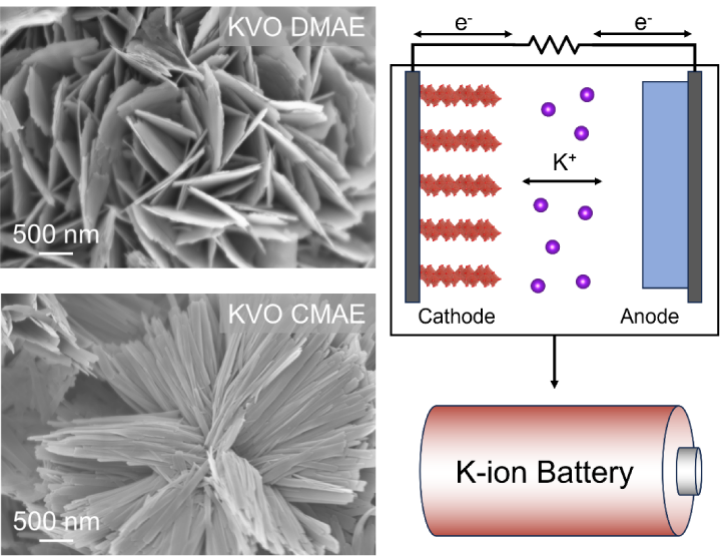

Bilayered vanadium oxides (BVOs) are promising cathode materials for beyond-Li-ion batteries due to their tunable chemistries and high theoretical capacities. However, the large size of beyond-Li^+^ ions limits electrochemical cycling and rate capability of BVO electrodes. Recent reports of MXene-derived BVOs with nanoscale flower-like morphology have shown improved electrochemical stability at high rates up to 5C in nonaqueous lithium-ion batteries. Here, we report how morphological stabilization can lead to improved rate capability in potassium-ion batteries (PIBs) through the synthesis and electrochemical characterization of MXene-derived K-preintercalated BVOs (MD-KVOs), which were derived from two V_2_CT_*x*_ precursor materials prepared using two different etching protocols. We show that the etching conditions affect the surface chemistry of the MXene, which plays a role in the MXene-to-oxide transformation process. MXene derived from a milder etchant transformed into a nanoflower MD-KVO with two-dimensional (2D) nanosheet petals (KVO-DMAE) while a more aggressive etchant produced a MXene that transformed into a MD-KVO with one-dimensional (1D) nanorod morphology (KVO-CMAE). Electrochemical cycling of the produced MD-KVOs after drying at 200 °C under vacuum (KVO-DMAE-200 and KVO-CMAE-200) in PIBs showed that electrochemical stability of MD-KVO at high rates improved through the morphological stabilization of 2D particles combined with the control of interlayer water and K^+^ ion content. Structure refinement of KVO-DMAE-200 further corroborates the behavior observed during K^+^ ion cycling, connecting structural and compositional characteristics to the improved rate capability. This work demonstrates how proper synthetic methodology can cause downstream effects in the control of structure, chemical composition, and morphology of nanostructured layered oxide materials, which is necessary for development of future materials for beyond-Li-ion battery technologies.

## Introduction

Lithium-ion batteries (LIBs) continue to play a dominant role in electrical energy storage, comprising 98.9% of the market share of battery energy storage in 2023.^1^ The light and mobile Li^+^ ion has enabled the commercialization of Li-ion batteries in a variety of applications but concerns over material cost and environmental/ethical sourcing of lithium stand to challenge the longevity of the technology as demand for energy storage continues to rise. Alternatives to lithium-based materials have been considered, with sodium and potassium being highly attractive substitutes. Both elements are orders of magnitude more abundant and more uniformly distributed in the Earth’s crust than lithium, which can address many future shortcomings in sourcing materials.^2^ In terms of electrochemical behavior, while the standard K/K^+^ redox couple has a higher potential than Li/Li^+^ (−2.936 V vs −3.040 V),^3^ in a common electrolyte solvent mixture like ethylene carbonate/dimethyl carbonate (EC/DEC), the K/K^+^ couple is −0.15 V against a Li/Li^+^ reference.^4^ This can lead to a larger operating voltage window for potassium-ion batteries (PIBs) and improve the energy density in full cell batteries. By comparison, the Na/Na^+^ redox couple, another Li-ion alternative, is more positive for both standard potential and organic solvent cases.^3^ The solvated K^+^ ion is also smaller than both solvated Li^+^ and Na^+^ ions which can improve ion diffusion and kinetics during cycling. The material choices for nonaqueous PIBs are similar to those for LIBs, as, unlike sodium, K^+^ ions have demonstrated intercalation into graphite,^4^ a widely used anode material in commercial LIBs. This compatibility, combined with the abundance and low-cost of potassium, underscores the growing interest in K-ion batteries as a promising alternative for sustainable energy storage.

To develop PIBs with high energy and power densities, it is essential to design and synthesize cathode materials that can effectively harness the electrochemical properties of K^+^ ions. Prussian blue analogues emerged as the first K-ion cathode material primarily due to their open structures that can accommodate large cations.^5−7^ Later, performance of polyanionic compounds^8−10^ and layered transition metal oxides^11^ as cathodes in PIBs were reported, typically in contrast with their performance in Li-ion systems. The layered transition metal oxides (TMOs) are of particular interest because, in addition to high potentials characteristic of ion intercalation, their 2D interlayer gallery provides well-defined diffusion channels for K^+^ ions, which can enable high energy and power performance in nonaqueous PIBs. Reports of layered TMOs as K-ion cathode materials have surged since Eftekhari and Ji’s 2016 review of potassium secondary batteries.^12^ K_*x*_MnO_2_,^13^ K_*x*_CoO_2_,^14^ K_*x*_Mn_*y*_Co_*z*_O_2_,^15^ and K_*x*_Ni_*y*_Mn_*z*_O_2_,^16^ to name a few, have been borrowed from traditional Li-ion cathode chemistry and show significant K^+^ ion storage and relatively high rate tolerance in nonaqueous K-ion systems. However, cobalt- and manganese-based oxides show limitations in their chemistry and structure for realizing higher performance cathode materials. Expanding the interlayer region of layered materials and utilizing transition metals that undergo multiple reduction steps can lead to improved charge storage capabilities.

Bilayered vanadium oxides (δ-V_2_O_5_·*n*H_2_O, or BVO) have been extensively studied as cathode materials for various Li-ion and beyond-Li-ion^17−20^ electrochemical systems. BVOs exhibit advantage of the V^5+^ chemistry, enabling multiple reduction steps during ion intercalation associated with the theoretical capacity for these cathode materials to over 400 mAh·g^–1^.^17^ Water molecules reside in the interlayer region of BVO and act as pillaring supports. For example, for δ-V_2_O_5_·1.6H_2_O where interlayer water was reported to form a uniform layer, the (001) *d*-spacing was determined to be 11.55 Å.^21^ Other pillaring species can also be introduced during the sol–gel synthesis of BVOs as a method of tuning the gallery size. Monovalent and multivalent cations modify the BVO (001) *d*-spacing in a direct relationship with the hydrated cation radius.^18,22^ Alkylammonium ions have also been intercalated for even greater interlayer expansions.^23^ The sol–gel processability of BVOs makes morphological tunability possible as well. 1D nanorods and nanobelts^24,25^ and 2D nanosheets^26−28^ have been synthesized and showed different diffusion- and nondiffusion-limited electrochemical behavior when cycled as cathodes in PIBs. These factors make BVO use in nonaqueous K-ion systems highly attractive.

Potassium-preintercalated BVOs (KVOs) are typically synthesized using three main methods. First, amorphous K_0.5_V_2_O_5_ is produced through a sol–gel process, where α-V_2_O_5_ is dissolved in the presence of H_2_O_2_ and a potassium source like KI, KCl, or K_2_CO_3_ at room temperature.^29^ Crystalline K_0.5_V_2_O_5_ or K_0.486_V_2_O_5_ were obtained by dissolution of α-V_2_O_5_ and potassium precursor in water followed by hydrothermal treatment at 200 °C for 24 h, resulting in 1D nanorods and nanobelts.^29−36^ When the sol–gel step is followed by hydrothermal treatment, nanostructured KVO forms with lower long-range order compared to crystalline KVO.^37−40^ This two-step synthesis process also enables the stabilization of interlayer water that can aid with structural stability of a KVO electrode during electrochemical cycling but can also cause parasitic processes leading to electrochemical degradation. It was shown that the interlayer water content can be tuned with low-temperature annealing.^18^ In terms of nonaqueous K-ion cycling, the crystal structure and morphology of KVOs affect the ion storage mechanism. Amorphous KVOs show rectangular cyclic voltammetry (CV) profiles, indicative of a nondiffusion-limited charge storage mechanism due to the lack of crystallinity.^29^ In contrast, highly crystalline KVOs exhibit distinct redox peaks that correspond to the intercalation and deintercalation of K^+^ ions into the KVO interlayer gallery.^30,34,35^ Nanostructured KVOs may display a combination of the two behaviors, depending on KVO particle morphology and interlayer water content.^29^ While more crystalline materials offer higher capacities, they often sacrifice cycling stability. Thus, optimizing synthetic parameters to obtain the KVO structure with high specific capacity and extended cyclability, especially at increased operating currents, is essential for advancing K-ion storage capabilities, thereby driving the development of PIBs forward.

2D transition metal carbides (MXenes) have emerged as promising precursors for synthesizing BVO polymorphs for cathode active materials in nonaqueous Li-ion,^41^ K-ion,^38,39^ and aqueous Zn-ion^42,43^ systems. The type of precursor used influences the arrangement of nanostructured particles, with α-V_2_O_5_ leading to randomly oriented 1D nanoparticles^30,34^ and V_2_CT_*x*_ MXene resulting in 3D ″nanoflower″-like agglomerates.^38,39,41^ Ridley et al. demonstrated the synthesis of MXene-derived BVOs with Li^+^, Na^+^, K^+^, Mg^2+^, and Ca^2+^ ion preintercalation that produced the particles with nanoflower morphology.^41^ Sun et al. adapted this synthesis approach to produce a MXene-derived KVO (MD-KVO) as a cathode material alongside a MXene-derived metal–organic framework (MOF) anode for K-ion full batteries. The MD-KVO exhibited the specific capacity of 73 mAh·g^–1^ in a half-cell configuration and 63 mAh·g^–1^ in the full cell.^38^ Yang et al. modified the synthesis of MD-KVO to incorporate Na^+^ ions along with K^+^ ions. In this configuration, the potassium ions prepopulate the electrochemically active sites in the interlayer region while the sodium ions act as support pillars that stabilize the BVO layered structure during K^+^ ion cycling.^39^ In each case, the MXene-to-oxide transformation leverages the chemical reactivity of MXene, which readily dissolves and oxidizes in the presence of water or hydrogen peroxide. However, the reactivity of MXene can be modified by tuning its surface chemistry.^44^ In lieu of such tunability, the MXene-to-oxide transformation process may vary depending on the specific method of MXene synthesis and has yet to be explored for MXene-derived BVOs.

**Scheme 1:**
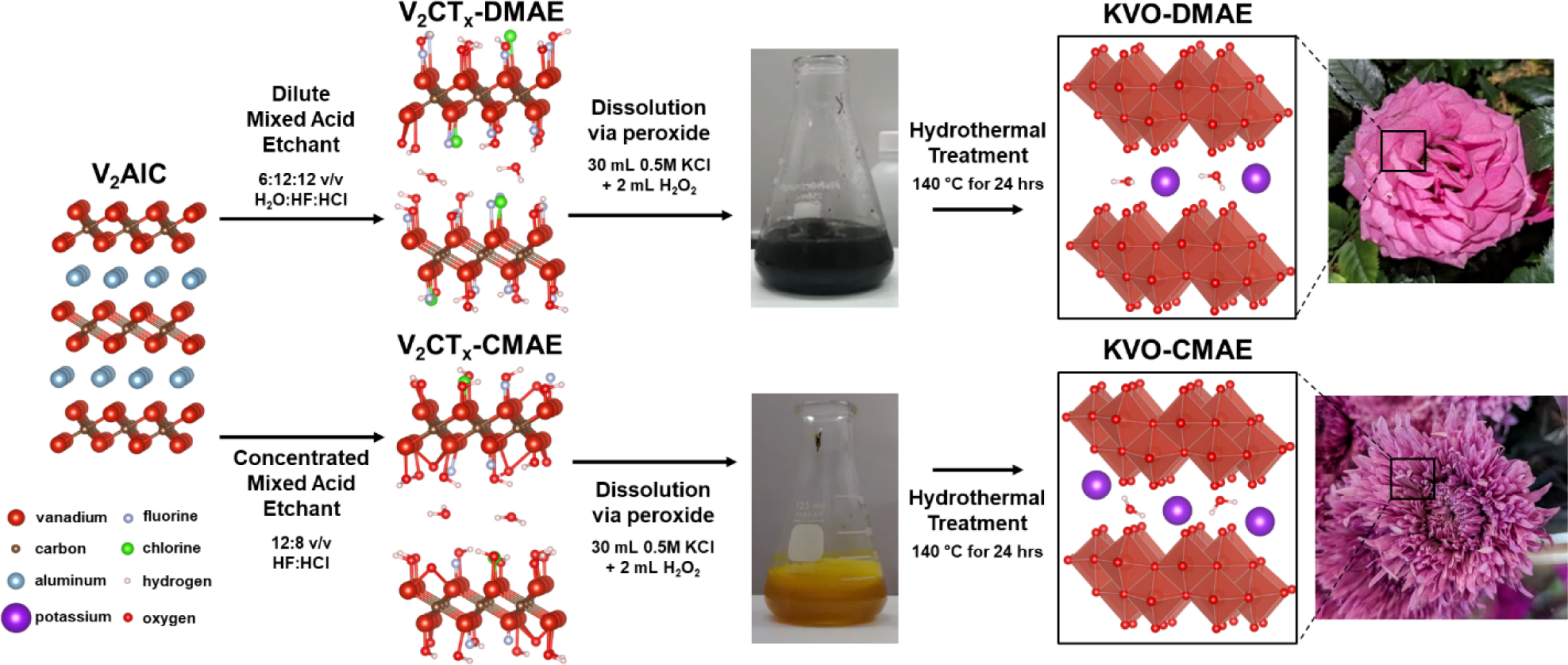
Schematic Illustration of the Three-Step Synthesis Process used to Produce KVO-DMAE and KVO-CMAE. V_2_AlC MAX Phase Is Etched to Obtain V_2_CT_x_ MXene Nanoflakes Using Two Different Etchant Compositions: Dilute Mixed Acid Etchant (DMAE, top) and Concentrated Mixed Acid Etchant (CMAE, bottom). Each V_2_CT_*x*_ MXene Is Then Dissolved in Water through the Addition of Hydrogen Peroxide in the Presence of K^+^ ions. Subsequent Hydrothermal Treatment of Each Solution Leads to the Formation of δ-K_*x*_V_2_O_5_·nH_2_O (KVO) with Distinctly Different Nanoflower-Like Morphologies That Resemble Roses (KVO-DMAE) and Chrysanthemums (KVO-CMAE). Photographs of the Flowers Were Taken by the First Author.

In this work, nanostructured MD-KVOs were synthesized using MXene precursors prepared with two different etching protocols and subsequently used as cathodes in nonaqueous K-ion cells. The synthesis pathways are outlined in Scheme 1. Two distinct V_2_CT*_x_* MXenes were synthesized with the use of a diluted mixed-acid etchant (DMAE) and a concentrated mixed-acid etchant (CMAE). These MXenes showed differences in surface terminations, which affected the dissolution process as MXenes reacted with water and hydrogen peroxide. After V_2_CT*_x_* MXene nanoflakes dissolution, induced by H_2_O_2_, in the presence of KCl, followed by hydrothermal treatment, δ-K_*x*_V_2_O_5_·*n*H_2_O powders were obtained from each MXene precursor and designated as KVO-DMAE and KVO-CMAE, reflecting the MXene precursor used in the synthesis. While the crystal structures of these materials were similar, differences appeared in the morphology of the samples and chemical composition of the interlayer regions. The potential window and drying conditions were optimized to identify the most suitable operating parameters for these materials as cathodes in nonaqueous K-ion cells. Drying conditions of 200 °C under vacuum (KVO-DMAE-200 and KVO-CMAE-200) and a potential window of 1.5–3.8 V were selected for further electrochemical analysis. X-ray total scattering and transmission electron microscopy (TEM) provided insights into the KVO-DMAE structure details allowing for better understanding of its charge storage properties in PIBs.

## Experimental Methods

### Synthesis of V_2_AlC MAX Powder

The synthesis of the MAX phase powder was adapted from a previous report.^41^ Vanadium powder (99.5%, −325 mesh, Alfa Aesar), aluminum powder (99.5%, −325 mesh, Alfa Aesar), and graphite (99%, −325 mesh, Alfa Aesar) were mixed in a 2:1.1:0.9 atomic ratio. The precursor materials were ball-milled with 10 mm zirconia balls (2:1 ball:powder ratio) in plastic jars at 50 rpm for 18 h. The powder mixture was then transferred to alumina crucibles, which were placed into a high-temperature tube furnace (Carbolite Gero). Ar gas (200 cm^–3^) was continuously flown through the furnace for 1 h prior to heating and during the entire annealing procedure. The temperature was increased to 1550 °C at 3 °C min^–1^, held for 2 h, and then cooled to room temperature at 3 °C min^–1^.

### Synthesis of V_2_CT_*x*_ MXenes

The synthesis of V_2_CT_*x*_ MXene nanoflakes was performed via a chemical etching technique of the V_2_AlC MAX powder. Two high-density polyethylene (HDPE) bottles were placed in oil baths on the hot plates, and Teflon stir bars were added to each bottle. To understand the effects of MXene surface chemistry on the oxide synthesis process, two etchants were prepared by mixing different ratios of deionized water, hydrochloric acid (HCl, 36.5–38%, Fisher Chemical), and hydrofluoric acid (HF, 48.5%–51%, Acros Organics). In one bottle, the first etchant, named “dilute mixed-acid etchant” or “DMAE”, consisted of a 6 mL:12 mL:12 mL volume ratio of water, HCl and HF, respectively, for every 1 g of MAX phase. After the DMAE was added, and the hot plate was set to 35 °C and 300 rpm, the MAX phase powder was slowly added over 5 min to reduce the buildup of initial reaction heat. This mixture was stirred for 5 days before washing.^41^ In the second bottle, a different etchant, named “concentrated mixed-acid etchant” or “CMAE”, consisted of a 12 mL:8 mL volume ratio of HF and HCl, respectively, for every 1 g of MAX phase. After the CMAE was added to the bottle, the hot plate was set to 50 °C and 300 rpm and the MAX phase powder was also added slowly over 5 min. This mixture was stirred for 3 days before washing.^44^

After etching was completed, each mixture was added to the 150 mL polypropylene bottle (Falcon) and filled with deionized water. These bottles were centrifuged at 3500 rpm for 5 min, and the supernatant was poured off to remove acidic solution. The sediment was redispersed in deionized water and this process was repeated until the pH reached the value of ≥ 5, after which the sediment was washed and filtered. The MXene powder was then collected, dried at 105 °C in air overnight, lightly ground in a mortar and pestle, and either used for oxide synthesis immediately or covered with Al foil and stored in a desiccator for future synthesis use. The two MXene precursors produced using DMAE and CMAE are referred to as “V_2_CT*_x_*-DMAE” and “V_2_CT*_x_*-CMAE”, respectively, throughout the manuscript.

### Synthesis of MXene-Derived KVOs

Chemically preintercalated δ-K_*x*_V_2_O_5_·nH_2_O samples were prepared through transformation of the two V_2_CT_*x*_ MXenes via a hydrogen peroxide-induced dissolution and hydrothermally assisted recrystallization process. First, 1.118 g of potassium chloride (KCl, 99.6%, Fisher Chemical) was dissolved in 30 mL of deionized water in a 125 mL Erlenmeyer flask with Teflon stir bar on a stir plate. Then, 300 mg of MXene powder (either V_2_CT_*x*_-DMAE or V_2_CT_*x*_-CMAE) was added to the flask while stirring, followed by the addition of 2 mL of hydrogen peroxide (H_2_O_2_, 30 wt %, Fisher Chemical). The mixtures were stirred for 2 h, during which the mixture changed colors depending on the MXene precursor. When V_2_CT*_x_*-DMAE was used, the color changed from black to a dark-green; when V_2_CT_*x*_-DMAE was used, the color changed from black to yellow. The underetched MAX phase remaining after MXene synthesis step settled to the bottom of the flasks, allowing the supernatant to be collected, avoiding any MAX phase impurity. The supernatant solutions were transferred to 23 mL Teflon liners (Parr Instruments Company), ∼15 mL of mixture in each. The liners were placed in acid digestion vessels (Parr instruments Company) and kept at 140 °C for 24 h. The resulting green precipitates were then washed, vacuum-filtered and dried at 105 °C in air overnight. The oxides derived from V_2_CT_*x*_-DMAE and V_2_CT_*x*_-CMAE in the presence of K^+^ ions are referred to as “KVO-DMAE” and “KVO-CMAE”, respectively. Separate samples were prepared with the additional step of annealing at 200 °C under vacuum and are referred to as “KVO-DMAE-200” and “KVO-CMAE-200”, respectively, throughout the manuscript.

### Materials Characterization

X-ray diffraction (XRD) patterns of the MAX, MXene, and oxide samples were obtained using a Rigaku Miniflex benchtop X-ray diffractometer with Cu Kα (λ = 1.54 Å) radiation. The patterns were collected using a step size of 0.02° and a step acquisition time of 0.7 s. Crystallinity analysis of XRD patterns was performed by calculating the area under the pattern of the crystalline peaks, calculating the area under the total pattern, and computing the fraction of crystalline area over total area to find the percent crystallinity. The full width at half-maximum (fwhm) was determined by measuring the horizontal distance between points for (001) peak at half of its maximum intensity. Scanning electron microscopy (SEM) images were obtained using a Zeiss Supra 50VP scanning electron microscope equipped with an in-lens secondary electron detector. SEM samples were sputter-coated with a thin layer of platinum/palladium on carbon to mitigate surface charging and improve image quality. Low and high magnification images were obtained using a beam accelerating voltage of 3 kV. The atomic absorption spectroscopy (AAS) analysis for potassium and vanadium was conducted using an AA-7000 atomic absorption spectrometer (Shimadzu, Japan). Calibration curves were developed using potassium standard solutions at concentrations of 0.1, 0.5, 1.0, and 2.0 μg/mL and vanadium standard solutions at 5, 10, 15, and 20 μg/mL. Both were prepared by diluting a 1000 μg/mL stock solution (Inorganic Ventures, USA) with analytical-grade water. For AAS sample preparation, approximately 10 mg of ground KVO-DMAE or KVO-CMAE powder was dispersed in a beaker containing analytical-grade deionized water, followed by the addition of one drop of 30 wt % H_2_O_2_ (Alfa Aesar, USA) to aid dissolution. The mixture was sonicated for 10 min and subsequently graduated in a 100 mL volumetric flask. The final AAS samples were prepared by further diluting 20 mL of the prepared solution to 100 mL. Potassium absorptions were measured using acetylene-air fuel, and vanadium absorptions were measured using an acetylene-N_2_O fuel with a flame optimization setup. Each cathode lamp was warmed up for 5 min prior to the atomic absorption measurements. Thermal weight-loss curves were obtained using a TA Instruments Q50 thermogravimetric analyzer (TGA). Weight-loss was evaluated from 25 to 1000 °C at a heating rate of 10 °C min^–1^. X-ray photoelectron spectroscopy (XPS) measurements were performed on a Physical Electronics VersaProbe 5000 using a monochromatic Al Kα source and Ar^+^ charge compensation. The high-resolution V 2p and O 1s, C 1s, F 1s, and Cl 2p spectra were taken at a pass energy of 23.5 eV with a step size of 0.05 eV. Peak fitting and data analysis were carried out using CasaXPS software. A Shirley background was used for spectra quantification. Spectra were calibrated to the C–V–O peak at 529 eV.^45^ Specific surface area, pore size distribution, and total pore volume were calculated using the Brunauer–Emmett–Teller (BET) method based on nitrogen adsorption measurements at 77 K with a Quantachrome Quadrasorb instrument. Prior to analysis, samples were degassed at 120 °C overnight to remove physisorbed species. X-ray total scattering experiments were performed at *National Synchrotron Light Source II* on the Pair Distribution Function (PDF, 28-ID-1) beamline at 74.46 keV (λ=0.1665 Å) with sample-to-detector distances of ∼ 1000 mm and ∼ 220 mm for PDF and XRD data sets, respectively. Samples were loaded in 1 mm diameter Kapton capillaries, and each measured for 10 min for the PDF data and 1 min for the XRD data on a PerkinElmer flat panel detector. Data was reduced using standard beamline data reduction protocols, and PDFs generated using PDFgetX3. The fitting of X-ray PDF data was conducted using *PDFgui* program,^46^ with the Q*damp* and Q*broad* values of 0.0371 and 0.0123, respectively, refined using data set from Ni standard (a = 3.525 Å). Bright-field scanning transmission electron microscopy (BF-STEM) images were acquired on an aberration-corrected Nion UltraSTEM 100 in the Center for Nanophase Materials Sciences (CNMS) at Oak Ridge National Laboratory (ORNL). The instrument was operated with a 100 kV accelerating voltage, a ∼31 mrad probe semiconvergence angle, and a probe current of ∼40 pA. The beam-sensitive nature of KVO-DMAE limited the total electron dose that could be applied to the material before lattice damage was observed to ∼2 × 10^4^ e^–^/Å^2^, which correspondingly limited the achievable spatial resolution compared to materials that are more robust under the electron probe.

### Electrode and Cell Fabrication

Electrodes were fabricated using a slurry casting method with a 7:2:1 mass ratio of the oxide sample, acetylene carbon black (Alfa Aesar), and polyvinylidene fluoride (PVDF, Kynar Flex, Arkema), respectively. The oxide and carbon black were first ground together in a mortar and pestle for 20 min and further mixed in a Flacktek DAC 150.1 FV-K speed mixer at 3000 rpm for 1 min to homogenize the mixture. In a separate container, the PVDF was dissolved in *n*-methyl-2-pyrrolidene (NMP, Acros Organics) and mixed in the Flacktek speed mixer. Once the PVDF was fully dissolved, the active material/carbon mixture was added to the PVDF solution and mixed once again. NMP was added to the slurry until the suspension was homogeneous. The final slurry was then spread on an aluminum foil (0.018 mm, Fisher Scientific) current collector and cast using a doctor blade set to 50 μm. The cast electrode was dried at 120 °C under vacuum for 24 h to remove any NMP and water trapped in the electrode film. Electrode disks were punched from the films using a 10 mm diameter punch. These disks were placed in a 105 °C vacuum oven for 24 h before being transferred to an argon-filled glovebox (MBraun). SEM images of the cast electrodes were analyzed to determine the particle radius distribution using the “Morphological Segmentation” function from the MorphoLibJ plugin^47^ in the Fiji software platform.^48^ Segmentation was performed with the “Input Image” parameter set to “Object Image”, the “Gradient Type” parameter set to “Morphological”, a “Gradient Radius” of 3, and the “Tolerance” of 6. The area of each segmented particle was calculated in Fiji and converted to an equivalent radius, assuming spherical particles with circular cross sections. Radii were binned from 0 to 40 nm with a bin width of 2 nm. Half-cells for electrochemical testing were assembled using 2032 coin cells. The prepared electrode disks acted as the working electrode. Potassium metal (Oakwood Chemical) was cleaned to remove its mineral oil storage medium, then rolled and punched into 12 mm diameter disks with a thickness of 100–300 μm, serving as both the reference and counter electrodes. This size of potassium metal anode ensures that the counter electrode does not limit the evaluation of the working electrode’s performance. Glass microfiber filters (Whatman) were used as separators, while 0.8 M KPF_6_ in 1:1 v/v EC/DEC solution served as the electrolyte.

### Electrochemical Characterization

To evaluate electrochemical stability of the MD-KVOs, materials were cycled in three potential windows: 2.0–3.7 V, 1.5–3.8 V, and 2.0–4.3 V. All potentials are reported with respect to the K/K^+^ reference electrode. Cyclic voltammograms were collected using a BioLogic VMP3 potentiostat. Cyclic voltammetry experiments were conducted using two sets of protocols. All materials were tested at a scan rate of 0.1 mV·s^–1^ and cycled for 5 cycles. Selected materials were subjected to scan rate-dependent cyclic voltammetry, cycling in the potential windows of 1.5–3.8 V at 0.1, 0.2, 0.4, 0.6, 0.8, and 1.0 mV·s^–1^ for 2 cycles, and the second cycle at each scan rate was reported. Peak currents were extracted from these profiles and fit according to two different models. First, b-value analysis^49^ was performed as a first-line test for charge storage analysis, modeled as
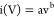
where i(V) is the current at a given voltage V, ν is the scan rate, and a and b are fitting parameters. A b-value of 0.5 indicates a diffusion-controlled process while a b-value of 1.0 indicates a nondiffusion- or surface-controlled process. Intermediate values indicate a mixed contribution of processes.^50^ A *k*_1_/*k*_2_-value analysis method^51^ was used to identify diffusion-limited vs nondiffusion-limited current contributions of these peaks based on the following model:
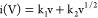
where i(V) is the current density at a given voltage, ν is the scan rate, and *k*_1_ and *k*_2_ are fitting parameters that correspond to the nondiffusion-limited and diffusion-limited current density contributions at a given voltage, respectively.

Galvanostatic rate capability testing was performed in the potential window of 1.5–3.8 V using current densities of 20, 50, 100, 200, and 20 mA·g^–1^ to evaluate the stability of MD-KVOs at varying operating currents.

## Results and Discussion

The formation of V_2_CT_*x*_-DMAE and V_2_CT_*x*_-CMAE MXenes was confirmed using XRD analysis (Figure S1). After etching, a new (002) peak emerges below 10° 2θ, indicative of the removal of Al and the insertion of protons and water molecules that expand the interlayer region. The (002) peak of V_2_AlC MAX phase has a *d*-spacing of 6.482 Å, which shifts to 9.705 Å for V_2_CT_*x*_-DMAE and 10.642 Å for V_2_CT_*x*_-CMAE MXene. Both V_2_CT_*x*_-DMAE and V_2_CT_*x*_-CMAE MXenes are multilayer powders that contain some unetched V_2_AlC. By comparing the relative intensities of the (103) peak of the remaining MAX phase and the (002) peak of the MXene, an estimate for the depth of etching can be evaluated. The V_2_CT_*x*_-CMAE XRD pattern has a less intense (103) peak compared to the V_2_CT_*x*_-DMAE XRD pattern, indicating a larger depth of etching and less V_2_AlC impurity in the V_2_CT_*x*_-CMAE sample. SEM images of these samples (Figure S2) confirm the successful etching of V_2_AlC. Interestingly, the morphology of the MXene particles at their edges vary depending on the etchant used. The high-magnification SEM image of V_2_CT_*x*_-DMAE (Figure S2d) shows a smooth edge while the high-magnification SEM image of V_2_CT_*x*_-CMAE (Figure S2f) reveals a more jagged edge. This difference in MXene edge appearance may be indicative of a trade-off in using a more concentrated etchant; the higher concentration of chlorine and fluorine ions can not only vigorously react with aluminum ions but also start to dissolve the vanadium atoms and overetch the material.^44^

**Figure 1:**
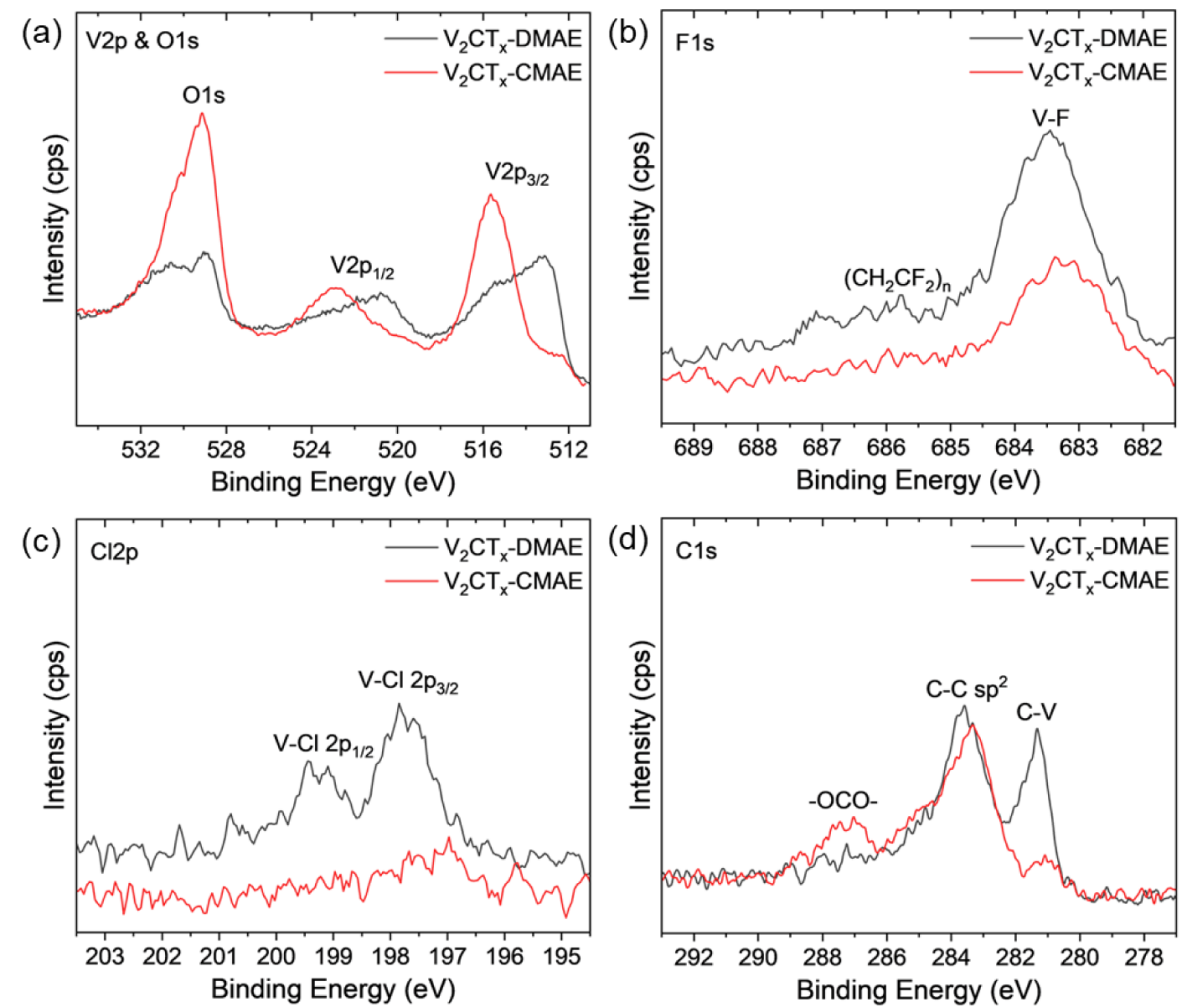
XPS spectra of V_2_CT_*x*_-DMAE and V_2_CT_*x*_-CMAE: (a) V 2p and O 1s, (b) F 1s, (c) Cl 2p, and (d) C 1s regions.

To understand the effects of the different etchants on the produced MXene chemistry, XPS spectra were examined to study the composition of V_2_CT_*x*_-DMAE and V_2_CT_*x*_-CMAE. V 2p, O 1s, C 1s, F 1s, and Cl 2p regions were identified for elemental analysis in the XPS survey scans (Figure S3); the atomic percentages of each element are summarized in Table S1. In the V 2p and O 1s region (Figure 1a), V 2p_3/2_, V 2p_1/2_, and O 1s peaks were identified for both V_2_CT_*x*_-DMAE and V_2_CT_*x*_-CMAE. The V_2_CT_*x*_-CMAE XPS spectrum showed shifts to higher energies as compared to the XPS spectrum of V_2_CT_*x*_-DMAE, indicating a higher average oxidation state of the vanadium atoms in V_2_CT_*x*_-CMAE. Analysis of the vanadium oxidation states by peak fitting (Figure S4 and Table S2) confirms the presence of the higher average oxidation state of vanadium in V_2_CT_*x*_-CMAE compared to V_2_CT_*x*_-DMAE. The oxidation states of vanadium in V_2_CT_*x*_-DMAE are more uniformly distributed while the oxidation states of vanadium in V_2_CT_*x*_-CMAE are more concentrated around 5+. The heightened oxidation state is accompanied by an increase in oxygen peak intensity, showing that CMAE incorporates more oxygen into V_2_CT_*x*_-CMAE during etching and is a more vigorous oxidant than DMAE. The fluorine content in the F 1s region (Figure 1b) and the chlorine content in the Cl 2p region (Figure 1c) are elevated in the V_2_CT_*x*_-DMAE XPS spectrum compared to the V_2_CT_*x*_-CMAE, indicating that the DMAE is a milder etchant that allows fluorine and chlorine to survive during etching compared to CMAE. Additionally, in the C 1s region (Figure 1d), the C–V peak around 281 eV is damped for V_2_CT_*x*_-CMAE while the C–C sp^2^ remains about the same, which may indicate the substitution of lattice carbon with oxygen.^52^ The rougher texture of the V_2_CT_*x*_-CMAE MXene particles visible in the SEM image (Figure S2) indicates that the surface terminations substitutional phenomenon, accompanying the etching process and damaging the edges of the MXene nanoflakes, correspond to the oxidation of the MXene lattice.

Hydrogen peroxide induced oxidation of V_2_CT_*x*_-DMAE and V_2_CT_*x*_-CMAE MXenes appears to occur via different transformation pathways. Visual monitoring of the solution color after H_2_O_2_ addition suggests partial dissolution of the MXene, yielding oxidized MXene particles suspended in a solution of vanadium ions, although the mechanism of the transformation is not yet understood.^53−55^ Timelapse video footage of the dissolution process for V_2_CT_*x*_-DMAE (Video S1) and V_2_CT_*x*_-CMAE (Video S2) shows the evolution of the solution color over 2 h. In the case of V_2_CT_*x*_-DMAE, the color changes from black (color of suspended MXene nanoflakes) to dark brown to red to a final color of dark green. The dissolution of V_2_CT_*x*_-CMAE follows a similar color change trajectory, with the exception that the final color is yellow. In both cases, the final liquids appear opaque, and once stirring is stopped, particles gradually settle out of suspension. Aqueous vanadium oxide chemistry is known to be correlated with the color of a given solution,^56^ therefore, to track the progression of the reaction we performed *in situ* pH measurements of the solutions during initial stage of MXene-to-oxide transformation (Figure S5). The pH of both dissolution reactions drops from the initial value of ∼3.0 to ∼ 1.5 within 1 min after H_2_O_2_ addition. Then, the pH starts to increase after around 20 min to ∼2.0, which is roughly correlated with a color change from black to red corresponding to dissolution of V_2_CT*_x_* nanoflakes. From this point, the reaction paths appear to diverge. The pH of the mixture containing V_2_CT_*x*_-DMAE continues to increase up to ∼3.5 and the color of the solution changes from red to yellow to dark green. The pH of the mixture containing V_2_CT_*x*_-CMAE stays at ∼2.0 and the color of the solution changes from red to yellow. Images of Tyndall scattering experiments applied to the solutions after 2 h of stirring (Figure S6) confirm the presence of suspended particles indicating that the nuclei for oxide growth have likely formed at this stage. From these images, the colors of the solutions are more apparent, as the liquid samples appear more translucent than in the videos. These suspensions, prepared after 2 h of stirring, were subsequently hydrothermally treated to promote oxide particle growth and form MD-KVOs.

**Figure 2:**
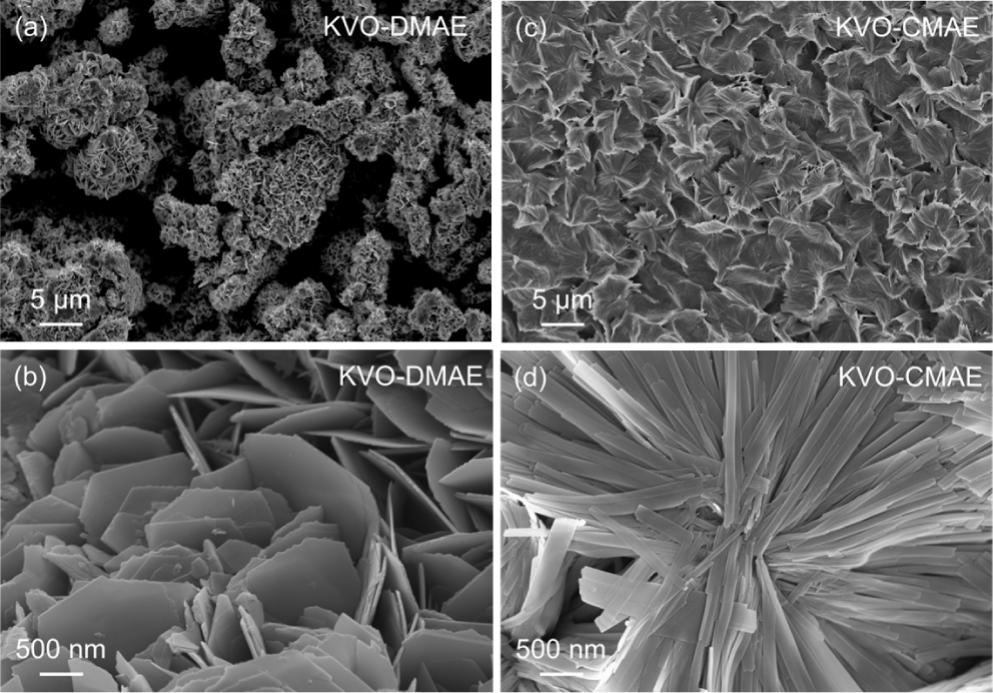
(a,c) Low- and (b,d) high-magnification SEM images of (a,b) KVO-DMAE and (c,d) KVO-CMAE.

SEM images of the produced MD-KVOs (Figure 2) reveal significant differences in the oxide morphology depending on the composition of the etchant used to prepare MXene precursors. The SEM images of KVO-DMAE present particles with 2D nanosheets intergrown to form 3D flower-like agglomerates resembling roses, which matches the morphology observed in the previous report.^41^ In contrast, the SEM images of KVO-CMAE show nanobelt particles emanating from the central point and forming agglomerates resembling the shape of a chrysanthemum. The central points from which 2D nanosheets in the case of KVO-DMAE and 1D nanobelts in the case of KVO-CMAE grow are believed to be the suspended oxidized MXene particles present in solution before hydrothermal treatment.

**Figure 3:**
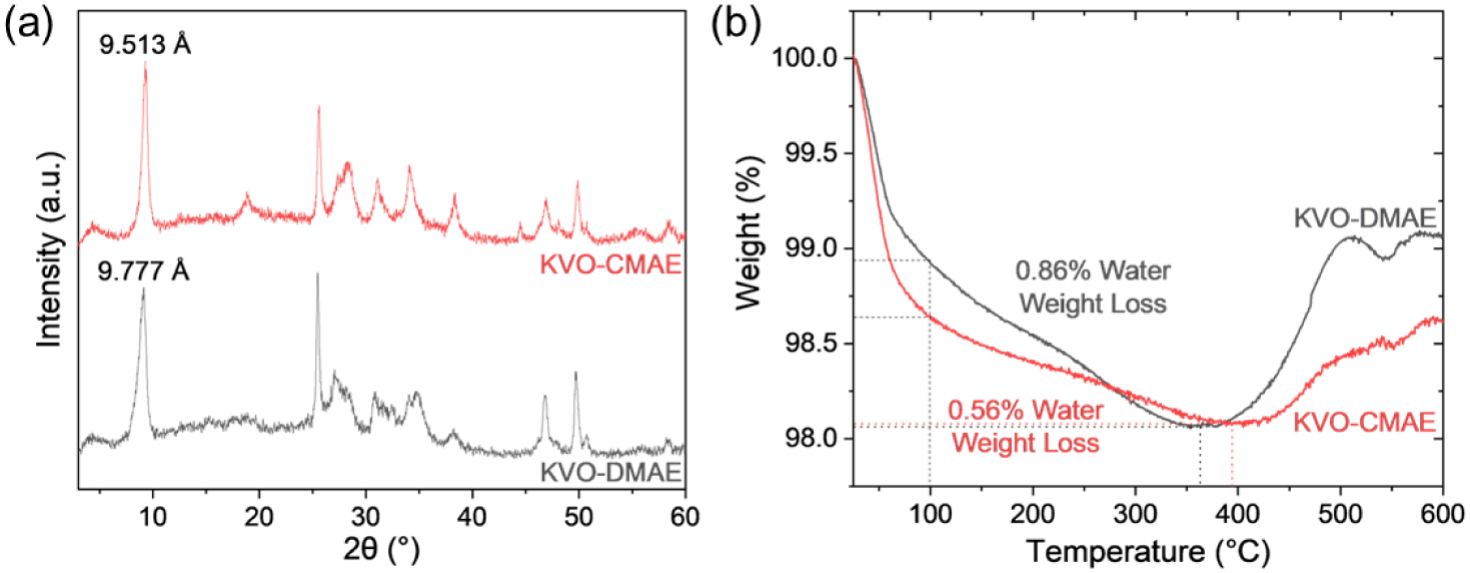
(a) XRD patterns and (b) TGA weight loss curves of KVO-DMAE and KVO-CMAE.

XRD patterns of the MD-KVOs (Figure 3a) indicate that KVO-DMAE and KVO-CMAE crystallized as layered phases in agreement with the δ-K_*x*_V_2_O_5_·*n*H_2_O structure.^41^ The intense and narrow peak around 9° 2θ corresponds to the (001) reflection of δ-K_*x*_V_2_O_5_·nH_2_O, and the (00*l*) peak series appears in the MD-KVO XRD patterns. The crystallinity of these samples was assessed to gain insight into their electrochemical charge storage behavior, as materials with higher crystallinity tend to exhibit greater capacity and stability. The percent crystallinity and fwhm of the (001) peaks were calculated from the XRD patterns of KVO-DMAE and KVO-CMAE (Figure S7). The percent crystallinity of KVO-DMAE and KVO-CMAE were found to be 43.0% and 49.9%, respectively, while the fwhm of the (001) peaks were 0.86 and 0.61, respectively. The higher crystallinity and smaller fwhm of (001) observed for KVO-CMAE indicate its superior crystallinity compared to KVO-DMAE. The *d*-spacing calculated from the position of (001) peak is 9.78 Å for KVO-DMAE and 9.51 Å for KVO-CMAE. The difference of 0.27 Å could possibly result from the differences in the interlayer K^+^ and water content. The K^+^ stoichiometry (x value in K_*x*_V_2_O_5_·*n*H_2_O) was estimated from AAS concentration analysis of MD-KVOs (Figure S8 and Table S3) giving chemical formulas of δ-K_0.46_V_2_O_5_·*n*H_2_O for KVO-DMAE and δ-K_0.41_V_2_O_5_·*n*H_2_O for KVO-CMAE. Water content (*n* value in K_*x*_V_2_O_5_·*n*H_2_O) was estimated from thermogravimetric weight loss curves (Figure 3c) by calculating the weight lost from 100 °C to the local minimum at ∼350–400 °C. 0.86% weight loss from interlayer water was estimated for KVO-DMAE and 0.56% for KVO-CMAE, which corresponds to *n* values of 0.096 and 0.062. Based on these measurements, the compositions of KVO-DMAE and KVO-CMAE can be estimated as K_0.46_V_2_O_5_·0.096H_2_O and K_0.41_V_2_O_5_·0.062H_2_O, respectively. The determined hydration degrees are consistent with the difference in *d*-spacings of 0.27 Å as the lower interlayer water content corresponds to a narrower interlayer region.^21^

**Figure 4:**
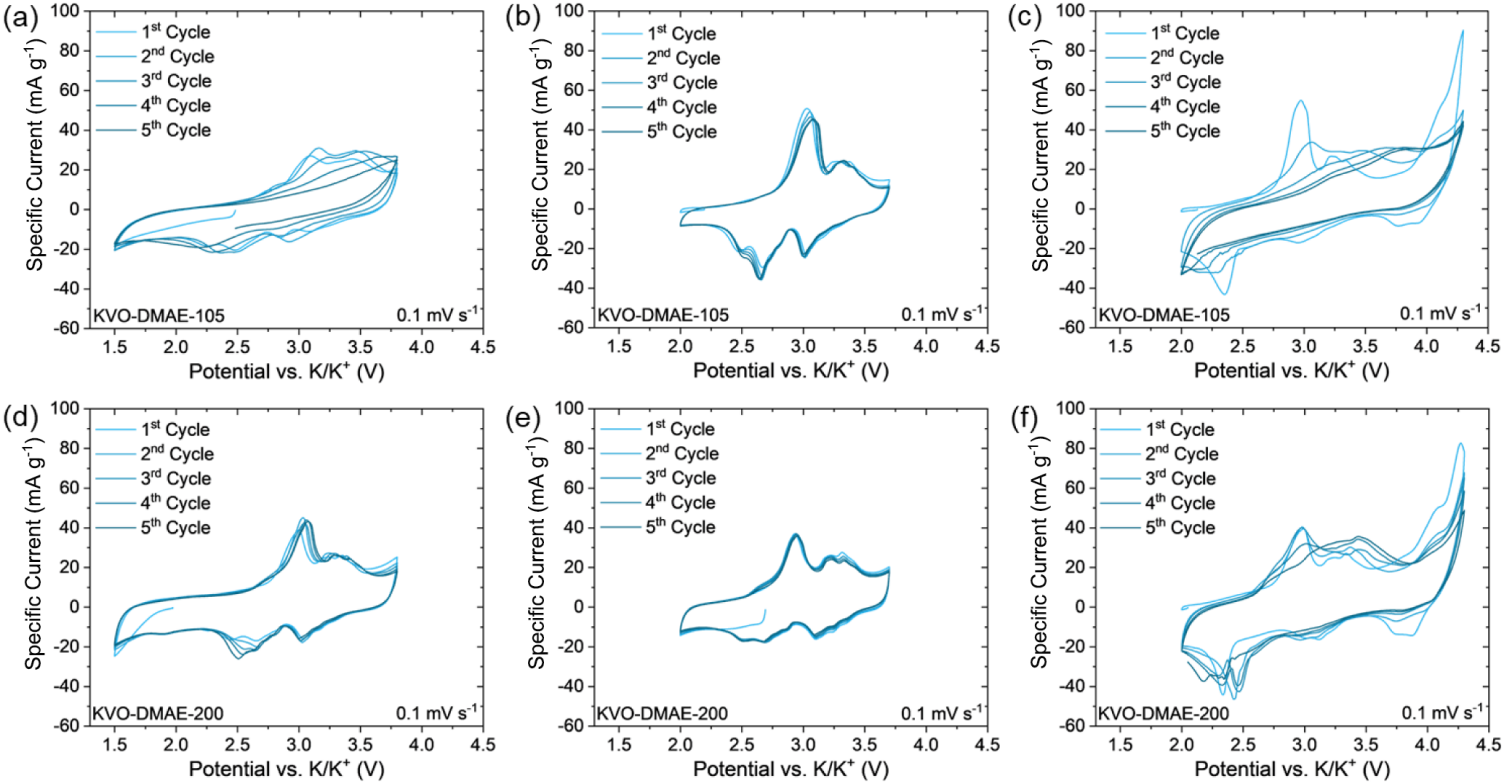
Effects of drying temperature and potential window on K^+^ ion cycling in cells containing KVO-DMAE electrodes: cyclic voltammetry (CV) profiles of (a−c) KVO-DMAE-105 cycled between (a) 1.5–3.8 V, (b) 2.0–3.7 V, and (c) 2.0–4.3 V, and (d–f) KVO-DMAE-200 cycled between (d) 1.5–3.8 V, (e) 2.0–3.7 V, and (f) 2.0–4.3 V.

It is established that the material drying conditions of the hydrated layered oxides and potential window during cycling play crucial roles in electrochemical stability of BVO polymorphs in nonaqueous intercalation batteries.^57,58^ In order to establish the proper conditions for MD-KVOs, KVO-DMAE was dried at 105 °C in air (called KVO-DMAE-105) and 200 °C under vacuum (called KVO-DMAE-200) and then cycled in K-ion cells in different voltage windows of 1.5–3.8 V, 2.0–3.7 V, and 2.0–4.3 V. KVO-DMAE was chosen due to its higher hydration degree implying that the change in drying conditions would show a more pronounced effect on cycling compared to KVO-CMAE. Corresponding cyclic voltammograms of KVO-DMAE in K-ion cells are shown in Figure 4. The cell containing KVO-DMAE-105 showed the most stable initial cyclability in the potential window of 2.0–3.7 V (Figure 4b). Expanding the potential window in either direction (Figure 4a,c) causes instability observed already in the first 5 cycles. The capacity decay is attributed to parasitic reactions related to the electrolyte degradation possibly caused by interlayer water in KVO structure. To mitigate the parasitic effect of interlayer water, drying at 200 °C under vacuum was explored. The XRD pattern of KVO-DMAE-200 (Figure S9a) confirmed the retention of the KVO structure, however a shift in the (001) peak position indicated the change in *d*-spacing from 9.78 Å to 9.47 Å, which agrees with the loss of interlayer water from 0.86% (*n* = 0.096) to 0.75% (*n* = 0.084) observed from TGA weight loss curves (Figure S9b). Interestingly, the CV profile of the K-ion cell containing the KVO-DMAE-200 electrode in the 2.0–3.7 V potential window (Figure 4e) indeed showed improved stability within the first 5 cycles however the capacity decreased compared to KVO-DMAE-105 (Table S5). This behavior could be attributed to the shrinkage of the interlayer region as water molecules are partially removed through the annealing and diminished charge screening effect known to be induced by interlayer water.^59^ The electrochemical stability of KVO-DMAE-200 was also improved in the 1.5–3.8 V potential window (Figure 4d) as compared to KVO-DMAE-105, however, expanding the potential window to 4.3 V (Figure 4e) still revealed rapid degradation. Based on these results, the 1.5–3.8 V and 2.0–3.7 V potential windows were selected for further electrochemical study of KVO-DMAE and KVO-CMAE. KVO-CMAE was dried at 200 °C under vacuum (called KVO-CMAE-200) to match the material processing conditions used for KVO-DMAE-200. The XRD pattern of KVO-CMAE-200 (Figure S9a) confirmed KVO structural retention, while the *d*-spacing changed from 9.51 Å to 9.35 Å due to the partial loss of interlayer water observed from TGA weight loss analysis (Figure S9b). The summary of the changes to (001) *d*-spacing and water weight loss are summarized in Table S4.

**Figure 5:**
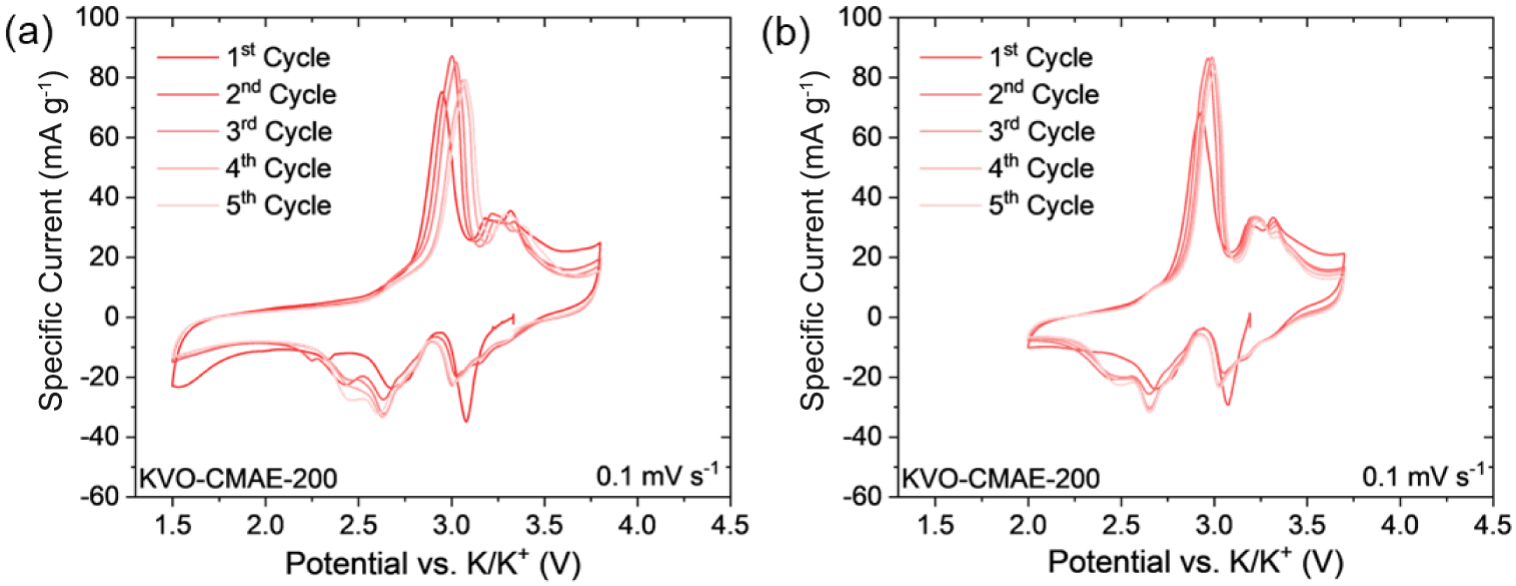
CV profiles of K-ion cells containing KVO-CMAE-200 electrodes in the potential windows of (a) 1.5–3.8 V and (b) 2.0–3.7 V.

CV profiles of cells containing KVO-DMAE-200 (Figure 4d,e) and KVO-CMAE-200 (Figure 5) electrodes reveal the electrochemical behavior of both materials in potential windows of 1.5–3.8 V and 2.0–3.7 V. These CV profiles showed similarities in shape with the CV profiles exhibited by the cell containing a K_0.5_V_2_O_5_ electrode reported by Deng et al.^30^ Three pairs of reversible cathodic/anodic peaks appear at 2.51 V/2.94 V, 2.68 V/3.22 V, and 3.10 V/3.33 V, with several reversible cathodic (3.36, 3.26, and 2.86 V) and anodic (2.64, 3.18, and 3.41 V) shoulders. The presence of these peaks and shoulders is attributed to the reversible K^+^ ion intercalation and deintercalation.^29,30^ The rectangular area in the CV profiles is larger for the cell containing the KVO-DMAE-200 electrode while the redox peaks are more pronounced in the case of the cell containing the KVO-CMAE-200 electrode. This is an indication of differences in the charge storage mechanism between the two electrodes, in agreement with the specific capacities calculated from the CV profiles (Table S5). During the charge on the second cycle, the KVO-DMAE-200 (KVO-CMAE-200) electrode exhibited specific capacities of 68.92 (72.09) and 51.15 (56.64) mAh g^–1^ in the 1.5–3.8 V and 2.0–3.7 V potential windows, respectively. On the fifth cycle, the discharge capacities increased in the 1.5–3.8 V window (up to 72.29 (74.53) mAh g^–1^ for the KVO-DMAE-200 (KVO-CMAE-200) electrode) while the capacities decreased in the 2.0–3.7 V window (down to 46.64 (54.62) mAh g^–1^ for the KVO-DMAE-200 (KVO-CMAE-200) electrode). Since cycling in the expanded 1.5–3.8 V window produced higher capacities that increased over 5 cycles, we chose these cycling conditions for further evaluation of the charge storage mechanism.

**Figure 6:**
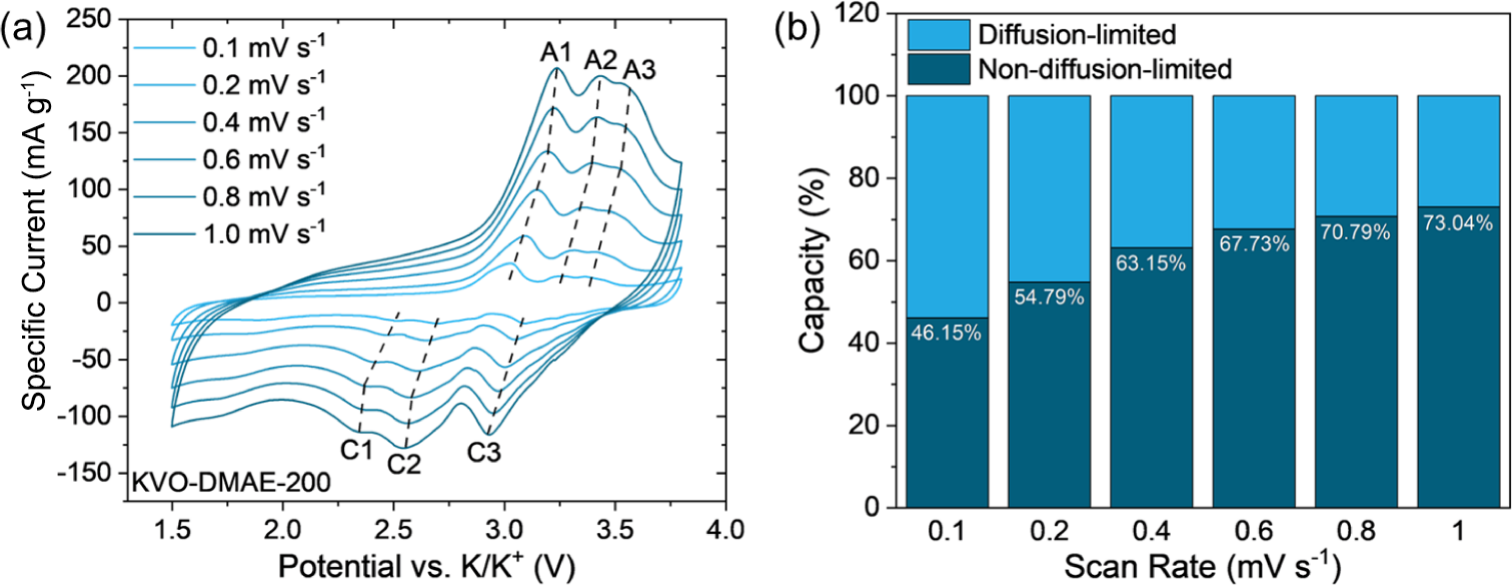
Charge storage mechanism analysis of KVO-DMAE-200. (a) scan rate-dependent CV curves of the K-ion cells containing KVO-DMAE-200 electrode from 0.1 mV/s to 1.0 mV/s and (b) summary of diffusion-limited and nondiffusion-limited capacity contributions at each scan rate. The listed percentage is the nondiffusion-limited contribution at a given scan rate.

The sweep-rate-dependent CV cycling from 0.1 to 1.0 mV s^–1^ showed that the KVO-DMAE-200 (Figure 6a) electrode was more stable at an increased sweep rate compared to the KVO-CMAE-200 electrode (Figure S10). The CV profiles of the cell containing the KVO-DMAE-200 electrode maintained their shape up to 1.0 mV s^–1^, with some shifting of the peaks. In contrast, the shape of the CV profiles for the cell containing the KVO-CMAE-200 electrode noticeably changed at 0.2 mV s^–1^, with the emergence of a single broad peak on discharge with no reverse peak on the corresponding charge step. This degradation in the CV profile shape indicates the poor promise of the KVO-CMAE-200 electrode for K-ion batteries, especially for high-power applications. This behavior contrasts with the previous crystallinity assessment, which suggested that KVO-CMAE may exhibit greater stability than KVO-DMAE, implying the presence of an alternative degradation mechanism. To confirm that this degradation is primarily driven by oxide properties, we analyzed the particle size distribution of the KVO-DMAE-200 and KVO-CMAE-200 electrodes using SEM images (Figure S11a,b) and the morphological segmentation function in Fiji software. The particle size distributions of KVO-DMAE-200 and KVO-CMAE-200 (Figure S11c,d) show similar profiles, indicating that the particle size distribution is comparable for both electrodes. This observation excludes electrode preparation as a factor in the rapid degradation of the KVO-CMAE-200, suggesting a material-property-driven degradation mechanism. Surface area measurements of the KVO-DMAE-200 and KVO-CMAE-200 powders determined using Brunauer-Emmitt-Teller (BET) theory reveal significant differences in porosity. The BET curves (Figure S12) show that the KVO-CMAE-200 powder has nearly twice the surface area of the KVO-DMAE-200 powder (35.81 m^2^·g^–1^ vs 20.30 m^2^·g^–1^). Additionally, the KVO-CMAE-200 powder has a smaller mode pore size (5.3 nm) compared to KVO-DMAE-200 (20.1 nm) and a larger pore volume (0.22 cc·g^–1^ vs 0.10 cc·g^–1^). These differences suggest that the smaller pores in KVO-CMAE-200 may allow greater electrolyte infiltration, accelerating the dissolution of the vanadium oxide, particularly at higher scan rates, where the additional polarization could provide more energy to overcome the energetic barrier of the dissolution reaction. These observations highlight the importance of proper selection of chemical etchant during the MXene-derived oxide synthesis process. As a result, charge storage mechanism analysis was infeasible for the KVO-CMAE-200 electrode. The KVO-DMAE-200 electrode was selected for further study. The *b*-value analysis was performed on the set of three cathodic peaks (C1:2.51 V, C2:2.68 V, C3:3.10 V, Figure 6a) and three anodic peaks (A1:2.94 V, A2:3.22 V, A3:3.33 V, Figure 6a). The calculated *b*-values (summarized in Figure S13a and Table S6) are in the range of 0.764–0.921, corresponding to a charge storage mechanism governed by a mixture of diffusion-limited and surface-controlled processes. Because these values lean closer to 1.0 rather than 0.5, this would indicate that surface-controlled processes are the dominant processes for these peaks. *k*_*1*_,*k*_*2*_-value analysis performed on the CV profiles (summarized in (Figures 6b, S13a and Table S6) showed agreement with the *b*-value analysis, indicating the charge storage mechanism is dominated by surface-controlled processes. While at low sweep rates, the nondiffusion-limited capacity contribution was lower than 50% at 0.1 mV s^–1^, at the higher sweep rates, this contribution begins to dominate with a 73.04% contribution at 1.0 mV s^–1^. The large nondiffusion-limited contribution can explain the stability of the KVO-DMAE-200 electrode at higher rates, as nondiffusion-limited processes involve fewer phase transformations of the active material during cycling.

The superior performance of the KVO-DMAE-200 over KVO-CMAE-200 can be attributed to two primary characteristics. First, the two-dimensional nanosheet morphology of the KVO-DMAE-200 sample likely provides a stabilization effect during K^+^ ion cycling, similar to chemically preintercalated MXene-derived BVOs in LIBs.^41^ The large faceted nanosheets can provide a larger and more continuous pathway for electron transport as compared to the one-dimensional nanorods of the KVO-CMAE-200 sample. This is reflected in electrochemical impedance spectroscopy (EIS) Nyquist plots of cells containing the electrodes (Figure S14); the KVO-DMAE-200 electrode shows a smaller semicircle diameter in the lower Z value region, which is indicative of a lower charge-transfer resistance. The improved transfer of electrons is likely to enable the observed advanced electrochemical behavior of KVO-DMAE-200, especially at higher rates. Second, the increased interlayer water content in the KVO-DMAE-200 sample can provide a water shielding effect that buffers the electrostatic interactions of the positively charged K^+^ ions and the negatively charged V–O bilayers, providing a charge screening effect on the K^+^ ions as they intercalate and deintercalated from the interlayer region.^60−62^ The tails of the EIS curves (Figure S14), associated with the Warburg element, also indicate better diffusion of K^+^ ions in the KVO-DMAE-200 electrode; the tails on the curves of the KVO-DMAE-200 electrode are shifted left compared to the KVO-CMAE-200 curves, which is indicative of a larger diffusion coefficient. Notably, while the charge screening effect was reported previously,^60,61,63^ here we for the first time observe this phenomenon for the rather small differences of hydration degrees. Improved rate capability exhibited by the KVO-DMAE-200 electrode is therefore attributed to better electron and ion transport.

**Figure 7:**
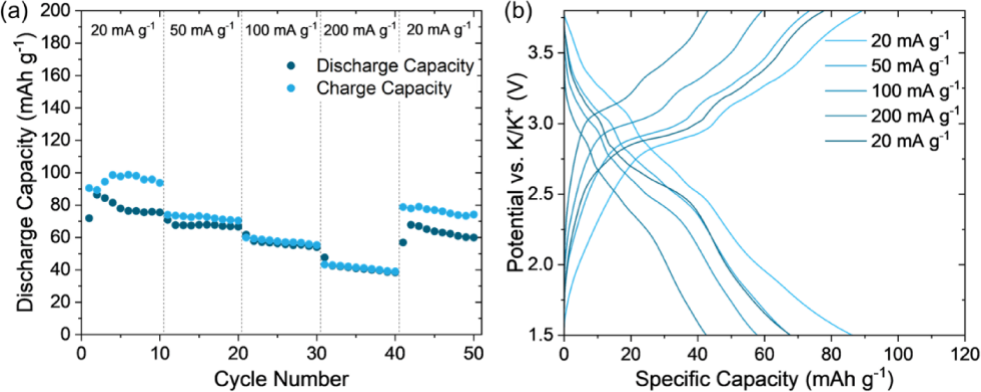
Galvanostatic rate capability testing of KVO-DMAE-200 at 20, 50, 100, 200, and 20 mA g^−1^: (a) discharge and charge capacities vs cycle number and (b) 2nd cycle charge/discharge profiles at each current density.

The rate capability testing of the cells containing KVO-DMAE-200 (Figure 7) and KVO-CMAE-200 (Figure S15) electrodes further supports the enhanced electrochemical behavior of the oxide obtained with the use of the dilute acid etching mixture to form V_2_CT*_x_* MXene precursor. The KVO-DMAE-200 electrode exhibits a maximum discharge capacity of 86.4 mAh·g^–1^ on the second cycle at the current density of 20 mA·g^–1^, and 39.0 mAh·g^–1^ at the 10th cycle at the current density of 200 mA·g^–1^. The specific capacity recovered to 67.8 mAh·g^–1^ after current density was returned to 20 mA·g^–1^. These capacities are in line with previous reports of KVOs in nonaqueous K-ion batteries (Table S8); KVO electrodes exhibited a maximum capacity between 62 and 90 mAh g^–1^ when tested in the 1.5–3.8 V potential window. In contrast, the KVO-CMAE-200 electrode delivered the maximum specific capacity of 69.1 mAh·g^–1^ at 20 mA·g^–1^, however the specific capacity dropped to negligible values at 200 mAh·g^–1^ (Figure S15a), in agreement with the observations in a sweep rate dependent CV cycling experiments. Additionally, KVO-DMAE-200 electrode showed good stability of the specific capacity for 10 cycles at a constant current density. The second cycle discharge/charge profiles shown by the cell containing the KVO-DMAE-200 electrode at each current density in the rate capability experiment (Figure 7b) show similar shapes with the plateaus appearing at 2.47, 2.67, and 3.05 V on discharge and 2.97, 3.22, and 3.35 V, in agreement with the positions of the peaks in CV profiles (Figure 4). For the KVO-CMAE-200 profiles (Figure S15b), the voltage plateaus disappear already at a current density of 50 mA·g^–1^. This observation agrees with the broadening of the peaks in CV profiles as higher sweep rates (Figure S10). Our results indicate the improved electrochemical stability in the KVO-DMAE-200 electrode at faster cycling, making it promising for high-power PIBs.

**Figure 8:**
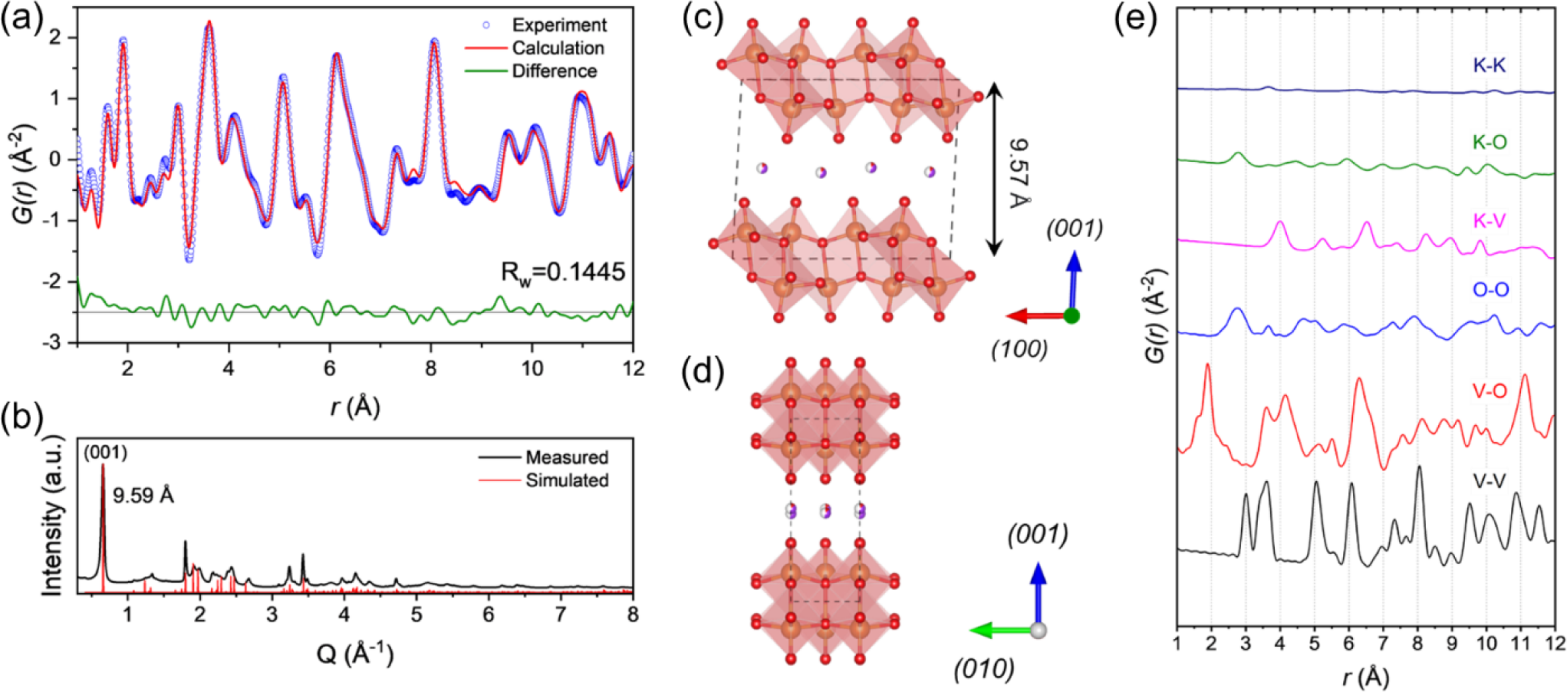
Synchrotron X-ray total scattering analysis of the KVO-DMAE structure. (a) PDF refinement of the X-ray total scattering data with associated goodness of fit (R_w_), (b) synchrotron XRD pattern and the simulated XRD obtained from the PDF refined structural model, shown in the (c) (100) ⊥ (001) plane and the (d) (010) ⊥ (001) plane, and (e) partial PDFs for each atomic pair involving K, V, and O.

To better understand what was driving the superior cycling performance of KVO-DMAE, we characterized its local atomic structure using X-ray total scattering. A simplified small-box K_0.5_V_2_O_5_ model (PDF Card #01-086-0347)^64^ was modified to include potassium and water content configured using experimentally obtained EDS and TGA results, respectively (K_0.40_V_2_O_5_·0.086H_2_O), where K^+^ ions and O atoms from interlayer water molecules occupied the same sites (Figure 8a). The experimental atomic pair distribution (PDF) pattern was fitted to this K_0.5_V_2_O_5_ model structure, which consists of a monoclinic unit cell with a *c* lattice parameter of 9.505 Å. The experimental PDF data fit well to the K_0.5_V_2_O_5_ model up to a radius of 12 Å, with an optimized goodness-of-fit value (R_w_) of 0.1445. The initial compositional values were set as a constant, so the composition remains the same after fitting. The spherical diameter parameter, which indicates the length scale of local average crystallographic order, was the primary variable considered for optimization. It was determined to be 55.52 Å, closely aligning with the particle average thickness of 45–50 Å measured from SEM images. Fitting results for the spherical diameters of 0, 60, and 120 Å were also determined (Figure S16). The refined *c* lattice parameter increased from 9.50 Å, characteristic of the model used, to 9.57 Å. This value is consistent with the (001) *d*-spacing of 9.59 Å observed in the synchrotron XRD pattern (Figure 8b). The structure parameters of KVO-DMAE obtained through this fitting process are provided in Table S7. The refined structure indicates that KVO-DMAE consists of two rows of edge-sharing VO_6_ octahedra, with water molecules and potassium ions confined in the interlayer region (Figure 8c,d). This atomic arrangement is further supported by the partial PDF decomposition of the fitted data (Figure 8e). Most of the G(r) signal corresponds to V–V and V–O bonding, representing the BVO structure, while the K–V and K–O signals are associated with the intercalated K^+^ ions. The interlayer water, represented by O atoms in the model, is correlated with the V–O, K–O, and O–O signals, but is convoluted by the O atoms in the V–O bilayer, so additional methods such as neutron scattering are necessary to determine the position of water molecules.

**Figure 9:**
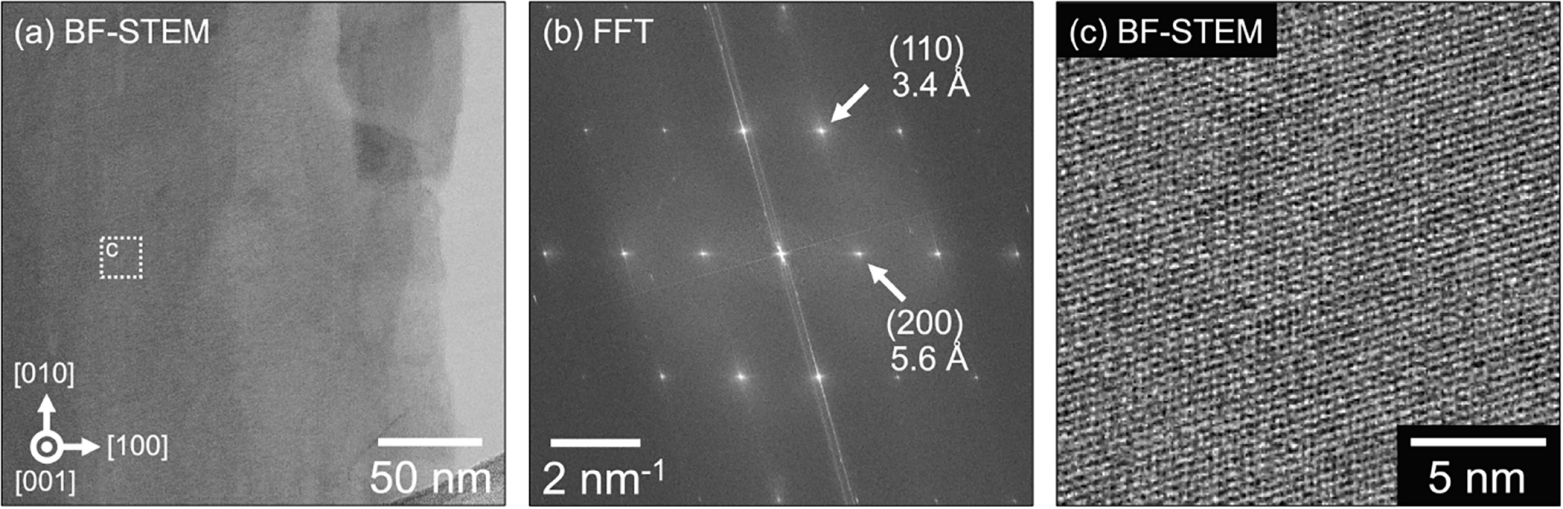
Atomic-scale imaging of KVO-DMAE by bright-field scanning transmission electron microscopy (BF-STEM). (a) BF-STEM image of the KVO-DMAE lattice acquired with the probe parallel to the [001] direction. (b) Fast Fourier Transform of the BF-STEM image in (a) showing lattice reflections perpendicular to the [001] direction. The experimentally measured lattice parameters match those of the PDF analysis to within ∼ 3–4%. (c) Zoomed region from (a) showing atomic-scale information about the KVO-DMAE lattice.

To further assess the accuracy of the PDF refinement structure, bright-field scanning transmission electron microscopy (BF-STEM) imaging of the KVO-DMAE lattice was performed. The unique 2D nanosheet morphology of these materials causes them to lie flat on the TEM grid with the [001] direction parallel to the STEM probe direction. This allows the intralayer lattice structure in directions perpendicular to [001] to be directly imaged. Despite the material’s beam-sensitive nature, which limits spatial resolution, Figure 9 shows an atomic-scale BF-STEM image of KVO-DMAE and the corresponding fast Fourier Transform (FFT), showing the presence of lattice reflections perpendicular to the [001] direction. Measurements of the (110) and (200) reflections gave lattice parameters of ∼3.4 Å and ∼5.6 Å, respectively, which are within ∼ 3–4% of the PDF refinement-generated values of 3.49 Å and 5.81 Å. These values show good agreement, demonstrating the accuracy of the PDF refinement structure. A zoomed region of Figure 9a showing the corresponding real space structure is shown in Figure 9c, where lattice planes perpendicular to the [001] direction can be directly observed.

The agreement between the structure obtained from the PDF refinement (Table S7) with the FFT of the BF-STEM images indicate the good accuracy of the composition estimated through EDS and TGA. The structure that was calculated also helps to explain the electrochemical behavior that was observed in K-ion batteries. The excellent rate capability implies efficient ion diffusion, which is likely enabled by the preintercalated K^+^ ions that act as interlayers pillars that predefine intercalation sites and diffusion pathway during operation. The interlayer water also likely provides a charge screening effect that buffers the K^+^ ion intercalation and further improves ion diffusion. Additionally, the 2D morphology of the KVO-DMAE-200 particles likely improves electron transport by providing a larger continuous pathway for electrons to travel as ions are entering and leaving the host material. This 2D morphology may also improve the stability at higher rates by suppressing the fraying of the KVO bilayers; the 1D nanobelts of KVO-CMAE-200 are more prone to delamination and/or dissolution as the interfacial area between two bilayers is smaller than for 2D KVO-DMAE-200.^65^ This combination of the morphological stabilization and interlayer chemistry led to the superior performance of KVO-DMAE-200 in PIBs, which was enabled by tuning of the etchant composition during the MXene synthesis process. The tunability in the use of MXene as the synthetic precursor is not limited to the etchant composition; solid-solution MXenes that incorporate two or more transition metals can also play a significant role in the physical and electrochemical properties of the oxide derivative.^66^ Our findings highlight the importance of a proper synthetic methodology that enables control of structure, chemical composition, and morphology that are necessary for the development of the next generation of beyond-Li-ion battery materials.

## Conclusions

In this study, we showed how the electrochemical performance of nanostructured V_2_CT_*x*_ MXene-derived K-preintercalated bilayered vanadium oxides (MD-KVOs) was dependent on the etching parameters used to synthesize the MXene precursors. Using a more concentrated mixed acid etchant during MXene synthesis produces MXene with a more oxidized and defective surface chemistry. This surface chemistry is directly related to the morphological stabilization and interlayer chemistry of MXene-derived K-preintercalated bilayered vanadium oxides, affecting the dissolution chemistry and subsequent crystallization during the MXene-to-oxide transformation process. KVO-DMAE crystallized into 2D nanosheets that contained twice as much interlayer water as the 1D nanorods of KVO-CMAE. This water content was further tuned by drying at 200 °C under vacuum, which led to better electrochemical stability of the MXene-derived oxides in extended potential windows in potassium-ion batteries (PIBs). When cycling in the 1.5–3.8 V potential window, KVO-DMAE-200 exhibited superior stability at higher rates compared to KVO-CMAE-200, indicated by a dominant nondiffusion-limited charge storage mechanism and lower charge transfer resistance. These characteristics were enabled by the morphological stabilization of the 2D nanosheets as well as the potential charge screening effect of the interlayer water. Pair distribution function refinement and bright-field scanning transmission electron microscopy both reveal a well-defined atomic arrangement of the constituent MD-KVO components, which corroborate the effects of the morphology and interlayer water on the electrochemical performance. These results demonstrate the vital role of the MXene synthesis parameters in the MXene-to-oxide transformation process. Tailoring the MXene chemistry toward desired structural, chemical, and morphological properties of MXene derivatives will not only enable superior materials for beyond-Li-ion batteries, but also pave the way for novel functionalities only possible using MXenes as precursors.

## Supporting Information Available

XRD patterns and SEM images of V_2_AlC MAX phase and V_2_CT_*x*_ MXene precursors; XPS survey spectra of MXene precursors with elemental analysis; XPS spectra fitting of V 2p region with oxidation state analysis based on V 2p_3/2_ region; time lapse videos showing the dissolution of MXene in the presence of hydrogen peroxide; *in situ* pH measurements during MXene dissolution; images of the Tyndall scattering effect after MXene dissolution but before hydrothermal treatment; AAS absorbance plots with potassium and vanadium concentrations analysis; XRD patterns and TGA curves of KVO-DMAE and KVO-CMAE before and after vacuum drying at 200 °C; table showing potential windows used to test cycling conditions of K-ion cells containing KVO-DMAE-105, KVO-DMAE-200, and KVO-CMAE-200 with specific discharge and charge capacities for selected cycles; scan rate-dependent CV curves of cell containing KVO-CMAE-200 electrode; *b*-value and scan rate-dependent CV curve analysis for KVO-DMAE-200 with the summary of *b*-values and diffusion-/nondiffusion-limited current contributions for each selected peak; EIS spectra of K-ion cells containing KVO-DMAE-200 and KVO-CMAE-200 electrodes; rate capability testing of K-ion cell containing KVO-CMAE-200 electrode; structure parameters of KVO-DMAE determined through PDF refinement; PDF refinement results with fixed spherical diameter values used during refinement; summary of synthesis conditions and electrochemical cycling parameters and performance of K-preintercalated bilayered vanadium oxide cathodes in nonaqueous K-ion cells reported in literature and obtained in this study (PDF)
Timelapse video footage of the dissolution process for V_2_CT_x_-DMAE (Video S1) (MP4)
Timelapse video footage of the dissolution process for V_2_CT_x_-CMAE (Video S2) (MP4)
