# Supplementary material for: MXene-Derived Potassium-Preintercalated Bilayered Vanadium Oxide Nanostructures for Cathodes in Nonaqueous K-Ion Batteries
Source: ACS Appl Nano Mater. 2025 Apr 7;8(15):7582–95. doi: 10.1021/acsanm.5c00175 (PMC12012740; doi:10.1021/acsanm.5c00175)
Supplement: Supplementary file 1 — an5c00175_si_001.pdf [file an5c00175_si_001.pdf]

SUPPORTING INFORMATION

MXene-Derived Potassium-Preintercalated Bilayered Vanadium Oxide Nanostructures for Cathodes in Non-Aqueous K-Ion Batteries

Timofey Averianov[1], Xinle Zhang[1], Ryan Andris[1], Daniel Olds[2], Michael J. Zachman[3], Ekaterina Pomerantseva[1*]

[1] Materials Electrochemistry Group, Department of Materials Science and Engineering, Drexel University, Philadelphia, Pennsylvania 19104, United States

[2] National Synchrotron Light Source II, Brookhaven National Laboratory, Upton, NY, 11973, USA

[3] Center for Nanophase Materials Sciences, Oak Ridge National Laboratory, Oak Ridge, TN 37831, USA

* Corresponding Author: ep423@drexel.edu

Keywords: chemically preintercalated bilayered vanadium oxides; MXene-derived oxides; MAX phase etchant composition; K-ion batteries; morphological stabilization; charge storage mechanism

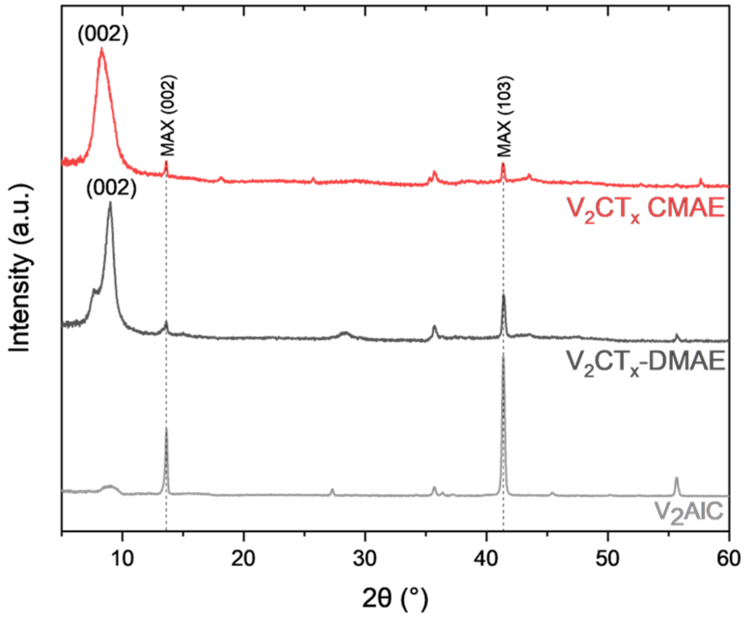

Figure S1. XRD patterns of V_2AlC MAX phase, V_2CT_x-DMAE MXene, and V_2CT_x-CMAE MXene.

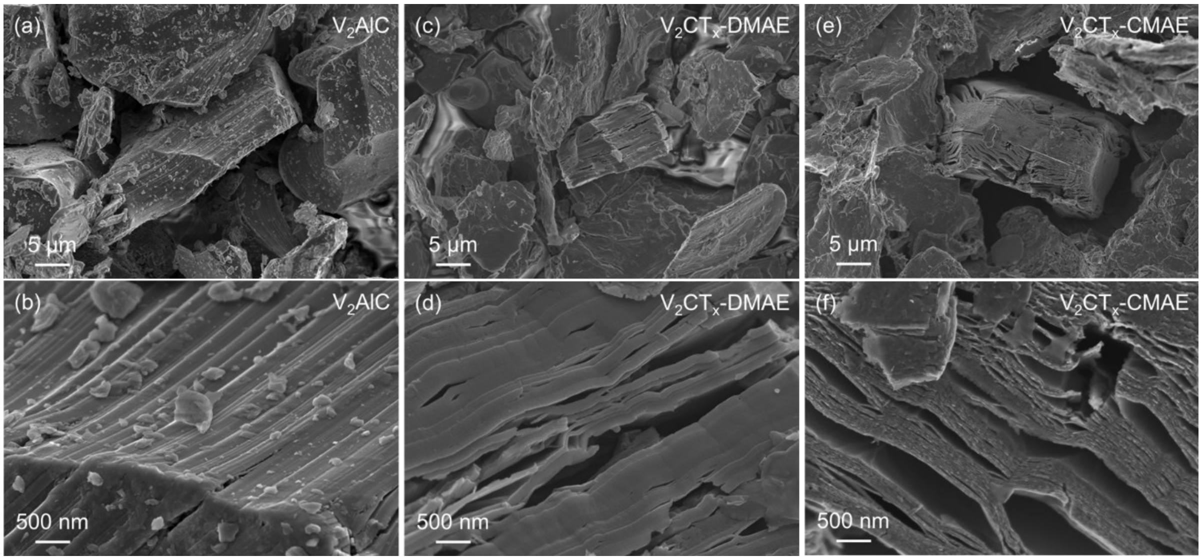

Figure S2. (a,c,e) Low- and (b,d,f) high-magnification SEM images of (a,b) V_2AlC MAX phase, (c,d) V_2CT_x-DMAE MXene, and (e,f) V_2CT_x-CMAE MXene.

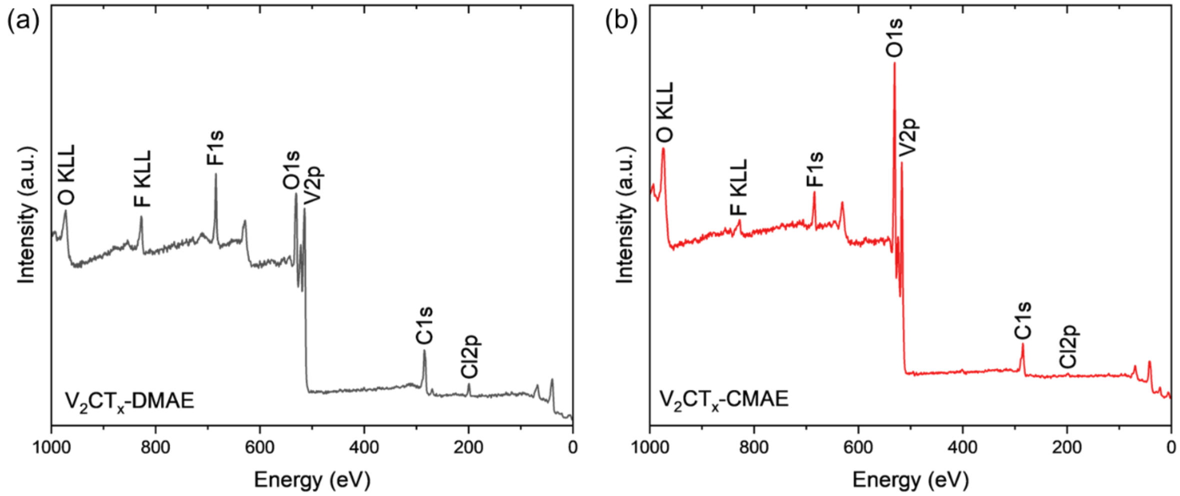

Figure S3. XPS survey spectra for (a) V_2CT_x-DMAE MXene and (b) V_2CT_x-CMAE MXene.

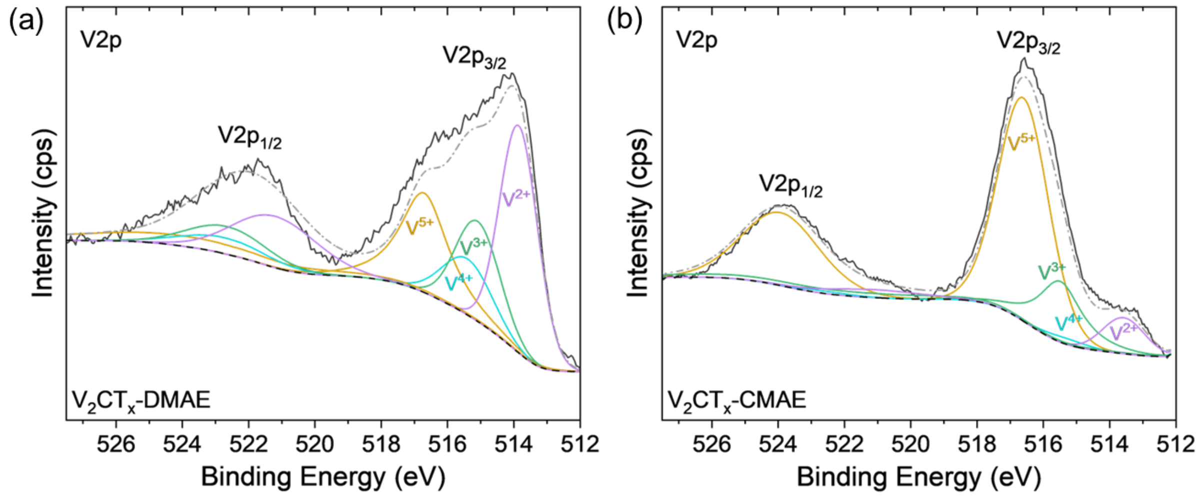

Figure S4. Fitted V2p region in XPS spectra of (a) V_2CT_x-DMAE and (b) V_2CT_x-CMAE.

Table S1. XPS analysis of elements for V_2CT_x-DMAE MXene and V_2CT_x-CMAE MXene.

Sample	V 2p (%)	C 1s (%)	O 1s (%)	F 1s (%)	Cl 2p (%)
V_2CT_x-DMAE	23.69	37.15	24.70	12.35	2.11
V_2CT_x-CMAE	22.01	23.80	46.02	7.70	0.47

Table S2. Calculated contributions of vanadium oxidation states in V_2CT_x-DMAE and V_2CT_x-CMAE from XPS analysis.

$V2p_{3/2}$	5+ (%)	4+ (%)	3+ (%)	2+ (%)
V_2CT_x-DMAE	26.09	13.02	20.66	40.23
V_2CT_x-CMAE	70.31	1.56	18.91	9.22

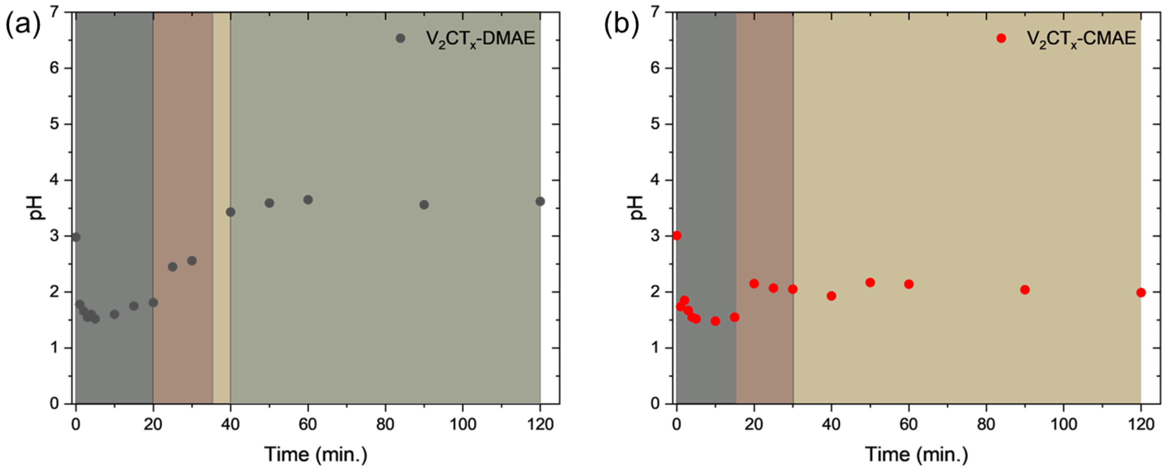

Figure S5. *In situ* pH measurements during the dissolution of (a) V_2CT_x-DMAE and (b) V_2CT_x-CMAE MXene nanoflakes via addition of H_2O_2 in the presence of KCl. Colors in the background correspond to the observed color of the solution at a given time (e.g. dark red solution was observed from 20 min to 35 min for V_2CT_x-DMAE).

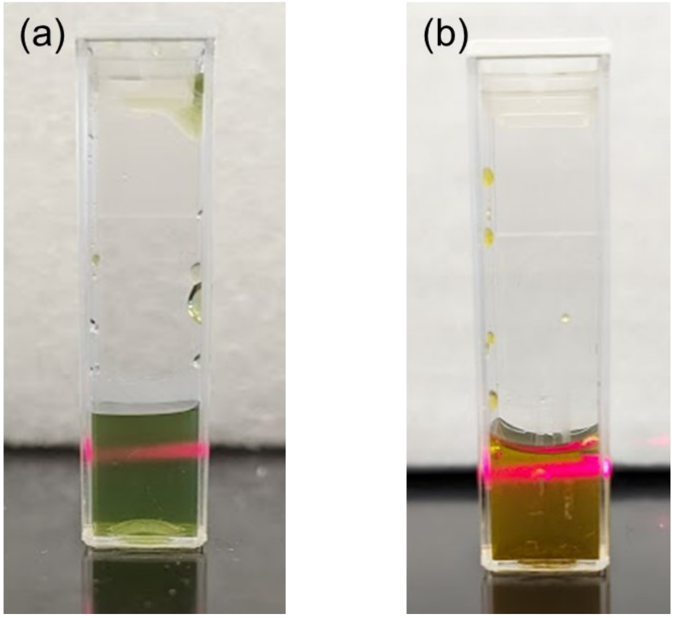

Figure S6. Images of Tyndall scattering experiments of the dissolved (a) V_2CT_x-DMAE and (b) V_2CT_x-CMAE before hydrothermal treatment.

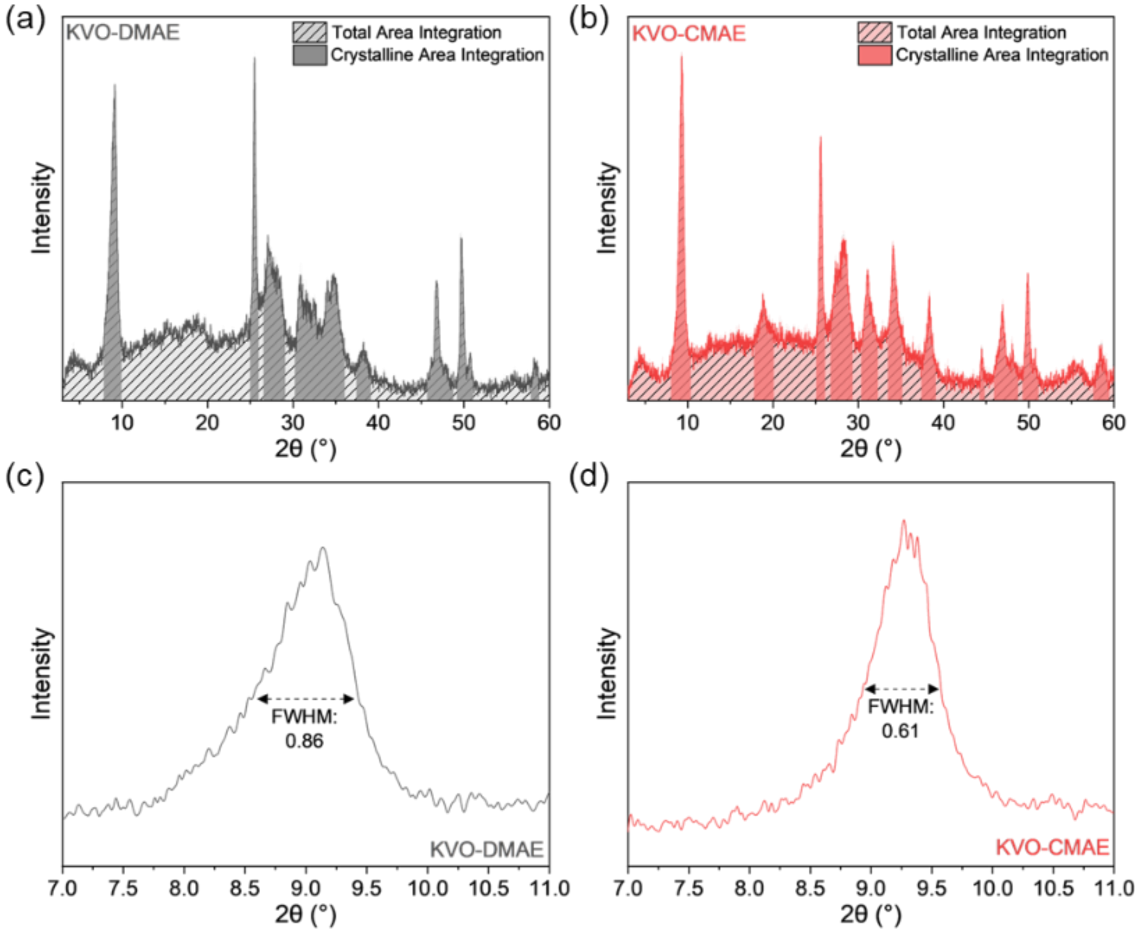

Figure S7. (a,b) XRD patterns with integrated area representations and (c,d) (001) peaks with FWHM analysis for (a,c) KVO-DMAE and (b,d) KVO-CMAE.

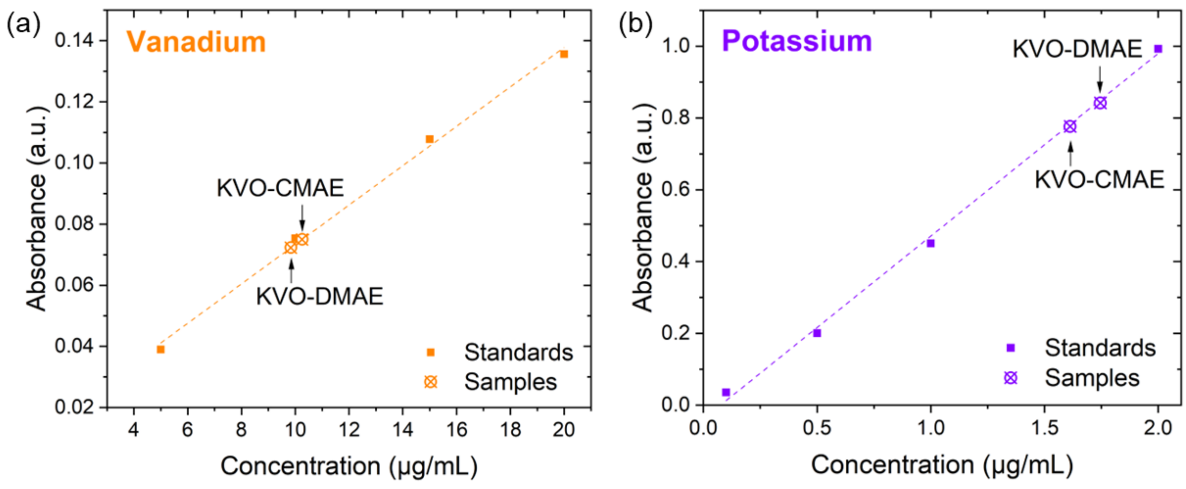

Figure S8. AAS absorbance plots for (a) vanadium and (b) potassium.

Table S3. Summary of vanadium and potassium concentrations and interlayer potassium content calculated from AAS measurements.

	**Vanadium concentration (μg/mL)**	**Potassium concentration (μg/mL)**	**K:V ratio**	**x in $K_xV_2O_5$**
KVO-DMAE	9.8386	1.7455	0.2311	0.4623
KVO-CMAE	10.2576	1.6125	0.2048	0.4096

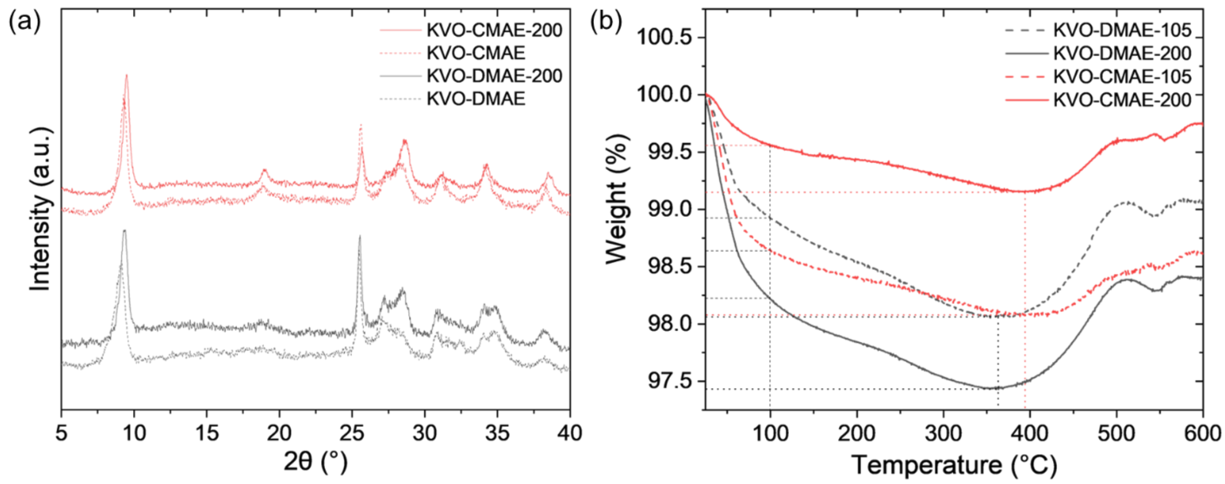

Figure S9. (a) XRD patterns and (b) TGA weight loss curves of KVO-DMAE and KVO-CMAE before and after 200°C vacuum drying.

Table S4. Summary of (001) *d*-spacings and weight loss associated with interlayer water content calculated from TGA weight loss curves for KVO-DMAE and KVO-CMAE before and after drying under vacuum at 200 °C.

Sample	KVO-DMAE-105	KVO-DMAE-200	KVO-CMAE-105	KVO-CMAE-200
(001) *d*-spacing (° 2θ)	9.72	9.47	9.53	9.35
TGA water weight loss (%)	0.81	0.75	0.56	0.41

Table S5. Summary of discharge and charge capacities determined from CV profiles exhibited by K-ion cells containing KVO-DMAE-105 and KVO-DMAE-200 electrodes.

Cycle #	Potential Window (V)	2nd Cycle Discharge Capacity (mAh g^{-1})	2nd Cycle Charge Capacity (mAh g^{-1})	5th Cycle Discharge Capacity (mAh g^{-1})	5th Cycle Charge Capacity (mAh g^{-1})
KVO-DMAE-105	1.5 – 3.8	79.06	73.50	57.10	44.22
	2.0 – 3.7	58.24	63.99	59.13	63.55
	2.0 – 4.3	72.22	131.55	53.41	94.76
KVO-DMAE-200	1.5 – 3.8	68.92	88.20	72.29	84.86
	2.0 – 3.7	51.15	69.05	46.64	64.07
	2.0 – 4.3	80.71	135.60	83.78	130.16
KVO-CMAE-200	1.5 – 3.8	72.09	102.90	74.53	96.76
	2.0 – 3.7	50.54	85.84	54.62	79.13

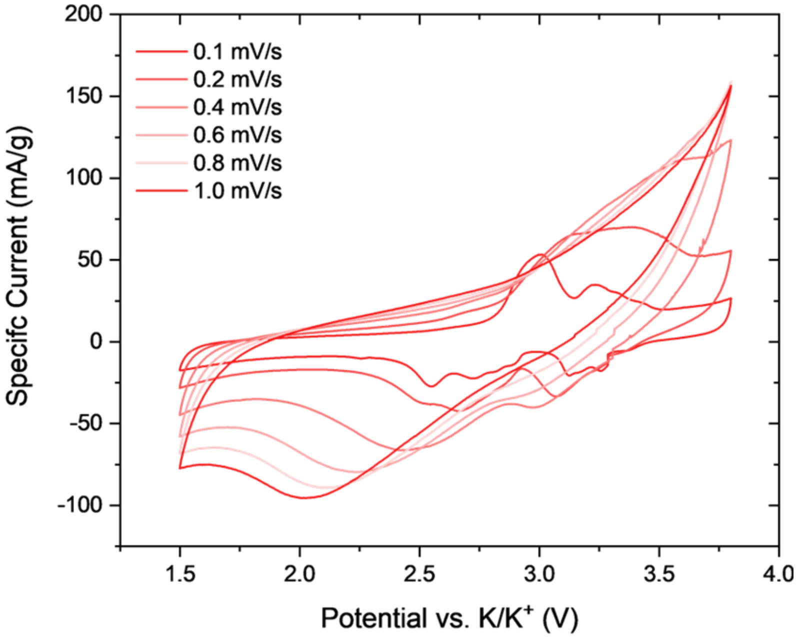

Figure S10. Scan rate-dependent CV curves of the K-ion cells containing KVO-CMAE-200 electrode from 0.1 mV s^{-1} to 1.0 mV s^{-1}.

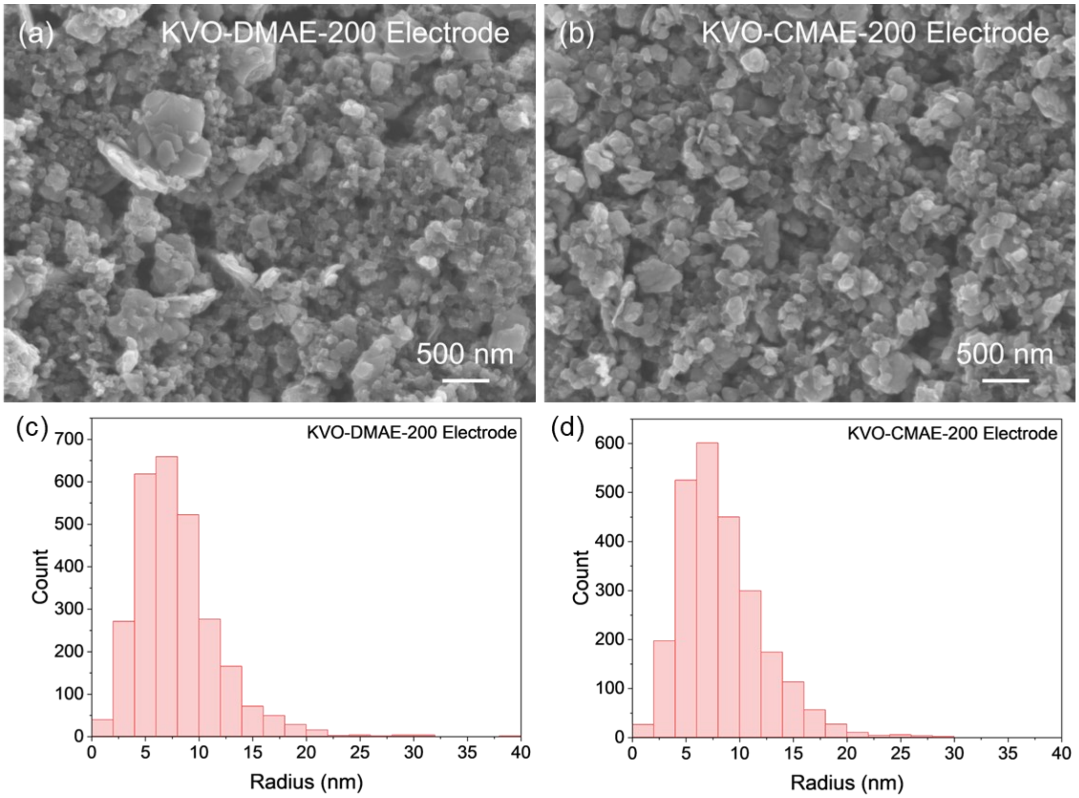

Figure S11. (a,b) SEM images and (c,d) particle radius distributions for (a,c) KVO-DMAE-200 electrode and (b,d) KVO-CMAE-200 electrode.

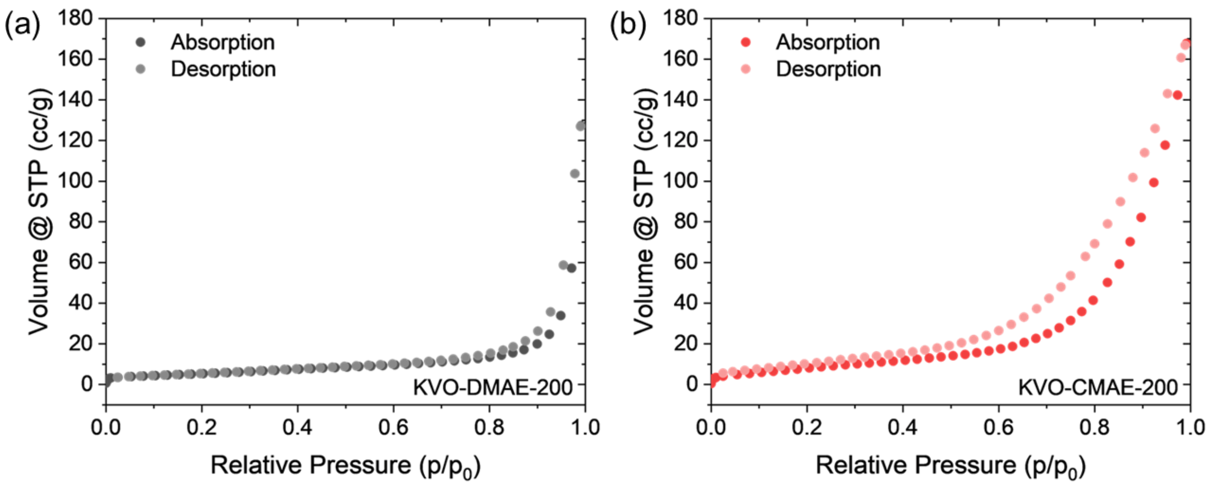

Figure S12. BET absorption/desorption curves for (a) KVO-DMAE-200 and (b) KVO-CMAE-200.

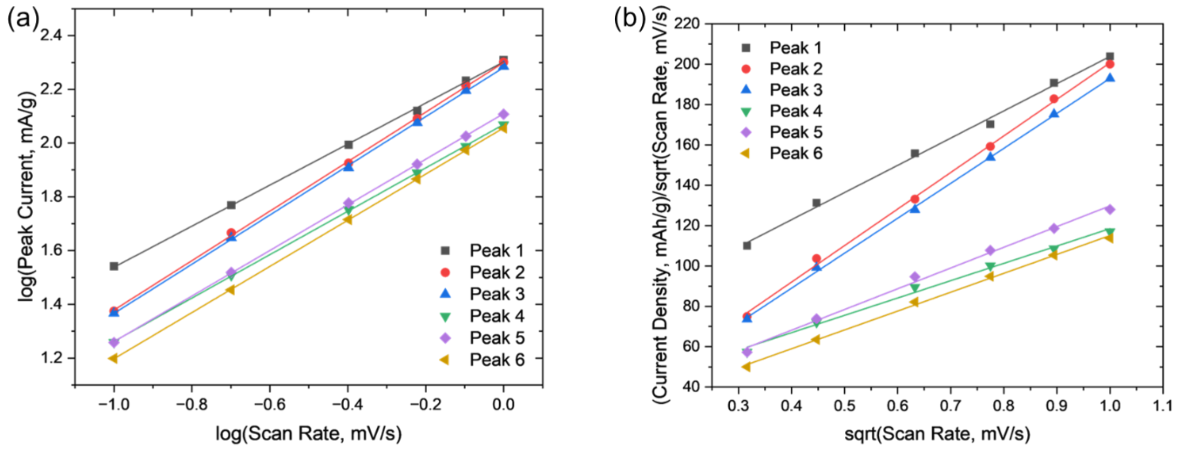

Figure S13. Application of (a) b-value and (b) scan rate-dependent CV curves analysis of the peaks identified in Figure 7a.

Table S6. List of b-values and diffusion-limited/non-diffusion-limited current contributions from potentiostatic rate capability analysis of KVO-DMAE-200.

Peaks	A1	A2	A3	C1	C2	C3
b-value	0.764	0.921	0.915	0.859	0.849	0.807
% diffusion-limited	33.831	9.748	10.207	18.739	20.863	27.520
% non-diffusion-limited	66.169	90.252	89.793	81.261	79.137	72.480

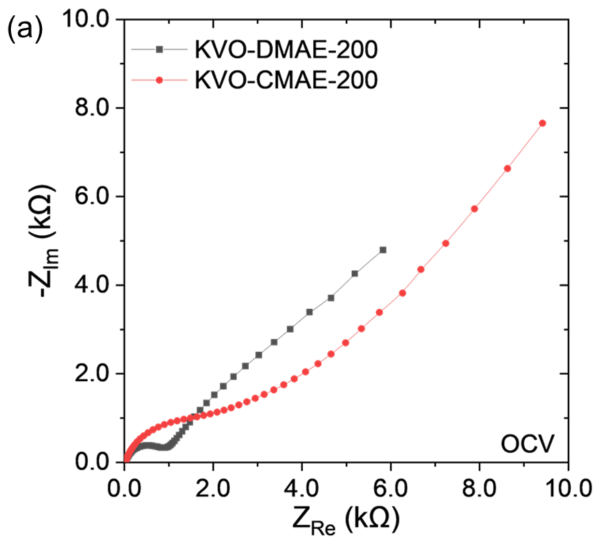

Figure S14. Electrochemical impedance spectra (EIS) of KVO-DMAE-200 and KVO-CMAE-200 electrodes at (a) OCV and (b) 0% state of charge (1.5 V vs. K/K^+).

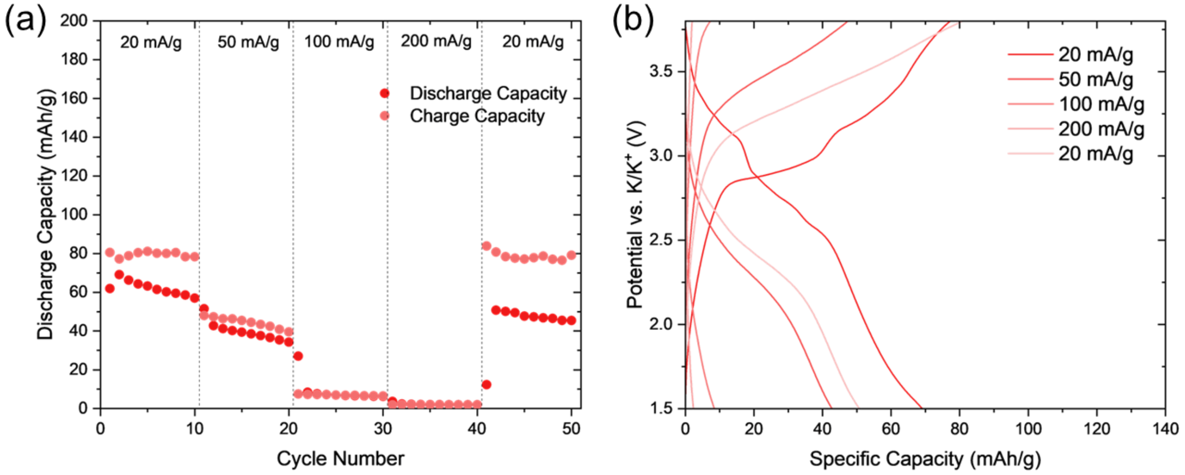

Figure S15. Galvanostatic rate capability testing of KVO-CMAE-200 at 20, 50, 100, 200, and 20 mA/g. (a) discharge and charge capacities and (b) 2nd cycle charge/discharge profiles at each current density.

Table S7. Structure parameters of KVO-DMAE determined via PDF refinement.

**a**	**b**	**c**	**β**
11.640000	3.661720	9.572730	92.992897

Atom	Label	Occ	a	b	c	U11	U22	U33	U12	U13	U23
V	V1	1	0.942094	0	0.154787	0.00267	0.00257	0.01478	0	0.0003	0
V	V2	1	0.237521	0	0.153682	0.00465	0.00329	0.00753	0	0.0003	0
O	Ow	0.133	0.614421	0	0.486761	0.03166	0.00603	0.00943	0	0.00069	0
K	K1	0.405	0.614421	0	0.486761	0.03166	0.00603	0.00943	0	0.00069	0
O	O1	1	0.405528	0	0.12911	0.01095	0.00001	0.01094	0	0.0004	0
O	O2	1	0.092309	0	0.09411	0.00922	0.01731	0.02939	0	0.0004	0
O	O3	1	0.770042	0	0.09631	0.01385	0.03098	0.00665	0	0.0004	0
O	O4	1	0.960275	0	0.320903	0.01102	0.0066	0.00246	0	0.00074	0
O	O5	1	0.223216	0	0.321294	0.01287	0.01696	0.01025	0	0.00069	0

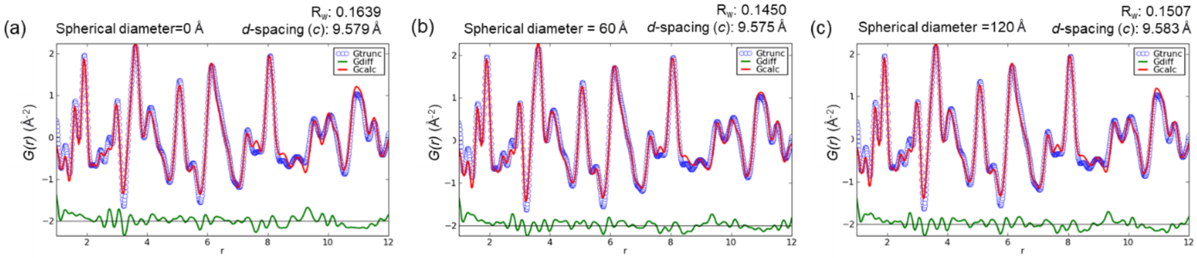

Figure S16. PDF refinement of the X-ray total scattering data with an applied fixed spherical diameter of (a) 0 Å, (b) 60 Å, and (c) 120 Å. The goodness of fitting (R_w) and interlayer distance (lattice constant "*c*") are shown in the figure.

Table S8. Comparison of performance of MD-KVOs and other KVO cathodes from literature.

Material	Drying Conditions	Electrolyte	Potential Window vs. K/K+	Max Capacity @ Current Density	Ref
$K_{0.5}V_2O_5$	80C for 24 hours	1.5M KFSI in 1:1 v/v EC:DEC	1.5 – 3.8 V	90 mAh/g @ 10 mA/g	[1]
δ-$K_{0.51}V_2O_5$	70C overnight	0.8M KPF6 in 95:5 v/v PC:FEC	2.0 – 4.5 V	131 mAh/g @ 30 mA/g*	[2]
$K_{0.486}V_2O_5$	"frozen-dry" followed by heating @ 5 C/min to 450C and maintained for 2h under nitrogen	0.8M KPF6 in 1:1 v/v EC:DEC	1.5 – 4.2 V	159 mAh/g @ 20 mA/g	[3]
$K_{0.5}V_2O_5$	80C for 24 hours	1.5M KFSI in 1:1 v/v EC:DEC	1.5 – 3.8 V	85.3 mAh/g @ 25 mA/g	[4]
$K_{0.5}V_2O_5$	80C under vacuum	0.8M KPF6 in 1:1 v/v EC:DEC	1.5 – 3.8 V	86 mAh/g @ 50 mA/g	[5]
nanocrystalline $K_{0.5}V_2O_5 \cdot 0.5H_2O$	80C for 12 hours under vacuum	1:2 w/w KFSI:TEP	2.0 – 4.5 V	104.5 mAh/g @ 20 mA/g	[6]
highly crystalline $K_{0.5}V_2O_5$	80C for 12 hours under vacuum	1:2 w/w KFSI:TEP	2.0 – 4.5 V	120.3 mAh/g @ 20 mA/g	[6]
amorphous $K_{0.5}V_2O_5$	80C for 12 hours under vacuum	1:2 w/w KFSI:TEP	2.0 – 4.5 V	~50 mAh/g @ 20 mA/g	[6]
$K_{0.5}V_2O_5$	freeze-dried	0.8M KPF6 in 1:1 v/v EC:DEC	2.0 – 4.1 V	78 mAh/g @ 20 mA/g	[7]
$K_{0.486}V_2O_5$	70C for 12 hours under vacuum	0.8M KPF6 in 1:1 v/v EC:DEC	1.5 – 3.8 V	61.92 mAh/g @ 20 mA/g	[8]
$K_xV_2O_5$ (MD-KVO)	100C for 12 hours	1.0M KPF6 in 1:1:1 v/v EC:DMC:DEC	1.5 – 3.7 V	73 mAh/g @ 100 mA/g	[9]
$K_{0.486}V_2O_5$	80C for 12 hours	3M KFSI in DME	1.8 – 4.0 V	90 mAh/g @ 20 mA/g	[10]
KVO	80C for 12 hours under vacuum	1.0M KPF6 in 1:1:1 v/v EC:DMC:DEC	1.5 – 4.3 V	110 mAh/g @ 100 mA/g	[11]
$K_{0.4}V_2O_5$	80C, then 300C for 1h	0.5M KPF6 in 1:1 v/v EC:DEC	1.5 – 4.2 V	98 mAh/g @ 20 mA/g	[12]
**$K_{0.400}V_2O_5 \cdot 0.086H_2O$ (KVO-DMAE)**	**105C for 12 hours in air, then 200C for 12 hours under vacuum**	**0.8M KPF6 in 1:1 v/v EC:DEC**	**1.5 – 3.8 V**	**86.4 mAh/g @ 20 mA/g**	**This work**
**$K_{0.417}V_2O_5 \cdot 0.041H_2O$ (KVO-CMAE)**	**105C for 12 hours in air, then 200C for 12 hours under vacuum**	**0.8M KPF6 in 1:1 v/v EC:DEC**	**1.5 – 3.8 V**	**69.1 mAh/g @ 20 mA/g**	**This work**

* cycled at 35C

References

1. Deng, L.; Niu, X.; Ma, G.; Yang, Z.; Zeng, L.; Zhu, Y.; Guo, L. Layered Potassium Vanadate $K_{0.5}V_2O_5$ as a Cathode Material for Nonaqueous Potassium Ion Batteries. *Adv. Funct. Mater.* **2018**, 28 (49), 1800670.
2. Zhu, Y.-H.; Zhang, Q.; Yang, X.; Zhao, E.-Y.; Sun, T.; Zhang, X.-B.; Wang, S.; Yu, X.-Q.; Yan, J.-M.; Jiang, Q. Reconstructed Orthorhombic V_2O_5 Polyhedra for Fast Ion Diffusion in K-Ion Batteries. *Chem* **2019**, 5 (1), 168-179.
3. Yuan, K.; Ning, R.; Bai, M.; Hu, N.; Zhang, K.; Gu, J.; Li, Q.; Huang, Y.; Shen, C.; Xie, K. Prepotassiated V_2O_5 as the Cathode Material for High-Voltage Potassium-Ion Batteries. *Energy Technol.* **2020**, 8 (1), 1900796.
4. Fan, Y.; Qu, Z.; Zhong, W.; Hu, Z.; Younus, H.A.; Yang, C.; Wang, X.; Zhang, S. Understanding the Effect of Interplanar Space and Preintercalated Cations of Vanadate Cathode Materials on Potassium-Ion Battery Performance. *ACS Appl. Mater. Interfaces* **2021**, 13 (6), 7377-7388.

5. Li, X.; Zhuang, C.; Xu, J.; Li, L.; Xu, T.; Dai, S.; Wang, X.; Li, X.; Wang, Y. Rational construction of $K_{0.5}V_2O_5$ nanobelts/CNTs flexible cathode for multi-functional potassium-ion batteries. *Nanoscale* **2021**, 13 (17), 8199-8209.
6. Niu, X.; Qu, J.; Hong, Y.; Deng, L.; Wang, R.; Feng, M.; Wang, J.; Zeng, L.; Zhang, Q.; Guo, L.; Zhu, Y. High-performance layered potassium vanadium oxide for K-ion batteries enabled by reduced long-range structural order. *J. Mater. Chem. A* **2021**, 9 (22), 13125-13134.
7. Deng, Q.; Wang, Y.; Yang, R.; Zhou, Y.; Luo, Z.; Liu, H.; Zhao, Z. Aluminum Fluoride Coating on Layered Vanadium-Based Cathode Materials with Enhanced K Storage Performance in the High Potential Range. *J. Phys. Chem. C* **2021**, 125 (39), 21359-21369.
8. Deng, Q.; Zhao, Z.; Wang, Y.; Wang, R.; Wang, J.; Zhang, H.; Feng, L.; Yang, R. A Stabilized Polyacrylonitrile-Encapsulated Matrix on a Nanolayered Vanadium-Based Cathode Material Facilitating the K-Storage Performance. *ACS Appl. Mater. Interfaces* **2022**, 14 (12), 14243-14252.
9. Sun, L.; Sun, J.; Zhai, S.; Dong, T.; Yang, H.; Tan, Y.; Fang, X.; Liu, C.; Deng, W.-Q.; Wu, H. Homologous MXene-Derived Electrodes for Potassium-Ion Full Batteries. *Adv. Energy Mater.* **2022**, 12 (23), 2200113.
10. Zhao, Y.; Liang, S.; Shi, X.; Yang, Y.; Tang, Y.; Lu, B.; Zhou, J. Synergetic Effect of Alkali-Site Substitution and Oxygen Vacancy Boosting Vanadate Cathode for Super-Stable Potassium and Zinc Storage. *Adv. Funct. Mater.* **2022**, 32 (32), 2203819.
11. Yang, H.; Li, Q.; Sun, L.; Zhai, S.; Chen, X.; Tan, Y.; Wang, X.; Liu, C.; Deng, W.-Q.; Wu, H. MXene-Derived Na^+-Pillared Vanadate Cathodes for Dendrite-Free Potassium Metal Batteries. *Small* **2024**, 20 (5), 2306572.
12. Oh, G.; Kansara, S.; Xu, X.; Liu, Y.; Xiong, S.; Hwang, J.-Y. Stabilizing Layered-Type $K_{0.4}V_2O_5$ Cathode by K Site Substitution with Strontium for K-Ion Batteries. *Adv. Funct. Mater.* **2024**, 34 (36), 2401210.
